# Sharing of human milk oligosaccharides degradants within bifidobacterial communities in faecal cultures supplemented with *Bifidobacterium bifidum*

**DOI:** 10.1038/s41598-018-32080-3

**Published:** 2018-09-18

**Authors:** Aina Gotoh, Toshihiko Katoh, Mikiyasu Sakanaka, Yiwei Ling, Chihaya Yamada, Sadaki Asakuma, Tadasu Urashima, Yusuke Tomabechi, Ayako Katayama-Ikegami, Shin Kurihara, Kenji Yamamoto, Gaku Harata, Fang He, Junko Hirose, Motomitsu Kitaoka, Shujiro Okuda, Takane Katayama

**Affiliations:** 1 Graduate School of Biostudies, Kyoto University, Sakyo-ku, Kyoto 606-8502 Japan; 2 Faculty of Bioresources and Environmental Sciences, Ishikawa Prefectural University, Nonoichi, Ishikawa 921-8836 Japan; 3 Graduate School of Medical and Dental Sciences, Niigata University, Chuo-ku, Niigata 951-8510 Japan; 4 Hokkaido Agricultural Research Center, National Agriculture and Food Research Organization, Sapporo, Hokkaido 062-8555 Japan; 5 Obihiro University of Agriculture and Veterinary Medicine, Obihiro, Hokkaido 080-8555 Japan; 6 Takanashi Milk Products Co., Ltd., Yokohama, Kanagawa 241-0023 Japan; 7 School of Human Cultures, The University of Shiga Prefecture, Hikone, Shiga 522-8533 Japan; 8 Food Research Institute, National Agriculture and Food Research Organization, Tsukuba, Ibaraki 305-8642 Japan

## Abstract

Gut microbiota of breast-fed infants are generally rich in bifidobacteria. Recent studies show that infant gut-associated bifidobacteria can assimilate human milk oligosaccharides (HMOs) specifically among the gut microbes. Nonetheless, little is known about how bifidobacterial-rich communities are shaped in the gut. Interestingly, HMOs assimilation ability is not related to the dominance of each species. *Bifidobacterium longum* susbp. *longum* and *Bifidobacterium breve* are commonly found as the dominant species in infant stools; however, they show limited HMOs assimilation ability *in vitro*. In contrast, avid *in vitro* HMOs consumers, *Bifidobacterium bifidum* and *Bifidobacterium longum* subsp. *infantis*, are less abundant in infant stools. In this study, we observed altruistic behaviour by *B*. *bifidum* when incubated in HMOs-containing faecal cultures. Four *B*. *bifidum* strains, all of which contained complete sets of HMO-degrading genes, commonly left HMOs degradants unconsumed during *in vitro* growth. These strains stimulated the growth of other *Bifidobacterium* species when added to faecal cultures supplemented with HMOs, thereby increasing the prevalence of bifidobacteria in faecal communities. Enhanced HMOs consumption by *B*. *bifidum*-supplemented cultures was also observed. We also determined the complete genome sequences of *B*. *bifidum* strains JCM7004 and TMC3115. Our results suggest *B*. *bifidum*-mediated cross-feeding of HMOs degradants within bifidobacterial communities.

## Introduction

The mammalian gut contains trillions of bacteria that can significantly influence host health through the production of metabolites and/or direct contact with host cells^1^. The gut microbial composition changes over time, with the most drastic changes occurring at the onset and termination of breast-feeding^2^. Recent studies have shown that the gut microbiota present early on in life has long-lasting effects on host health^3,4^; therefore, it is important to understand how the gut microbiota is established during infancy. Bifidobacteria are well-known as first colonisers of human intestines, and generally account for more than 50% (in many cases, more than 70%) of the total bacterial population in the gut of breast-fed infants^5–7^. A bifidobacteria-rich gut microbiota is correlated with a decreased incidence of diarrhoea, allergy, and atopic dermatitis^8,9^. Moreover, it has been shown that bifidobacteria can promote antitumor immunity^10^, reduce serum cholesterol levels^11^, and increase folate availability in the intestine^12^. Formation of a bifidobacteria-rich microbiota is thus considered to be important for the establishment of a life-long healthy gut environment in humans.

Human milk oligosaccharides (HMOs) is the collective term for oligosaccharides with a degree of polymerisation of ≥3 in breast milk. They are the third most abundant solid component in breast milk after lactose and lipids, but have no nutritional value for infants because of their resistance to pancreatic digestion^13,14^. Interestingly, mothers produce HMOs in the mammary glands with a high energy expenditure (one ATP is theoretically consumed by one glycosyltransferase reaction). Several groups, including our own, have shown that infant gut-associated bifidobacterial species, including *B*. *breve*, *B*. *bifidum*, *B*. *longum* subsp. *longum* (*B*. *longum*), *B*. *longum* subsp. *infantis* (*B*. *infantis*), and in rare case *B*. *pseudocatenulatum*, are equipped with sets of genes coding for enzymes dedicated to the degradation of HMOs^6,15–18^. Interestingly, within the gut community, these HMO-degrading enzymes are essentially limited to the above-mentioned bifidobacterial species^16,19,20^. Therefore, it is highly likely that HMOs serve as selective nutrients for these bacteria to prevail in the gut ecosystem. Indeed, we recently showed that the gene encoding lacto-*N*-biosidase (LnbX), an important HMO-degrading enzyme in *B*. *longum*, is enriched in the stools of exclusively breast-fed infants compared with those of mixed-fed infants^7^. It should be noted that the occurrence of *lnbX* in *B*. *longum* is strain-dependent, and less than half of the genome-sequenced *B*. *longum* strains contain the gene. These results strongly suggest that HMOs serve as a selective pressure for the formation of the gut microbiota, which could be driven by HMO-mediated symbiosis between certain bifidobacteria and humans.

**Figure 1:**
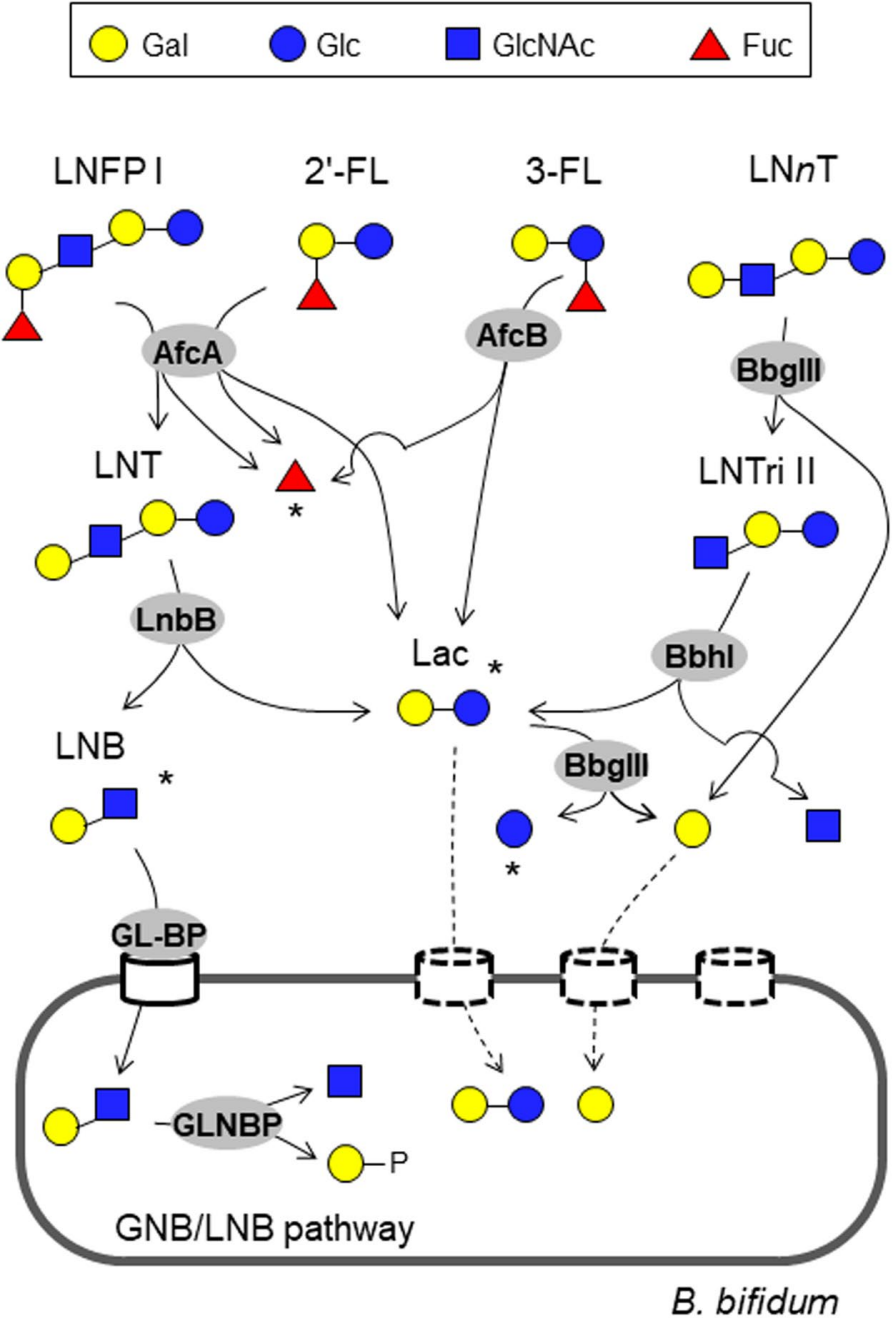
Schematic representation of the minimum enzymatic set required to degrade different sugar linkages found in neutral HMOs by *B*. *bifidum* (See also Table 1). Sugars are depicted according to the nomenclature committee of the Consortium for Function Glycomics. LNFP I (Fucα1-2Galβ1-3GlcNAcβ1-3Galβ1-4Glc), 2′-FL (Fucα1-2Galβ1-4Glc), 3-FL (Galβ1-4(Fucα1-3)Glc), and LN*n*T (Galβ1-4GlcNAcβ1-3Galβ1-4Glc) are shown as representatives of the different sugar linkages. AfcA: 1,2-α-l-fucosidase; AfcB: 1,3-1,4-α-l-fucosidase; LnbB: lacto-*N*-biosiadse; BbgIII: β-1,4-galactosidase; BbhI: β-*N*-acetylglucosaminidase; GL-BP: galacto-*N*-biose/lacto-*N*-biose I-binding protein of ABC transporter; GLNBP: galacto-*N*-biose/lacto-*N*-biose I phosphorylase. Dashed lines indicate pathways that have not been identified in *B*. *bifidum*. Degradants that accumulate in the spent media during culture in the presence of HMOs are indicated by asterisks (See Fig. 3 and Table S3).

Infant gut-associated bifidobacteria have evolved two different strategies to degrade HMOs^16^. The first is extracellular hydrolase-dependent, while the second is oligosaccharide transporter-dependent. In the former case, HMOs are hydrolysed extracellularly into mono- and disaccharides by cell wall-anchored secretory glycosidases, and the liberated sugars are then incorporated inside the cells (Fig. 1, described below), while in the latter case, HMOs with a degree of polymerisation of ≥3 are directly imported into the cells by ATP-binding cassette (ABC) transporters, and the resulting oligosaccharides are hydrolysed intracellularly by exoglycosidases^16^. Although our understanding of the HMO degradation pathway in bifidobacteria is incomplete, it appears that *B*. *bifidum* and LnbX-positive *B*. *longum* use extracellular hydrolases, while *B*. *breve*, *B*. *infants*, and LnbX-negative *B*. *longum* rely on oligosaccharide transporters^21^. We previously showed that the four infant gut-associated bifidobacterial species do indeed use the relevant glycosidases and transporters to assimilate HMOs *in vitro* by analysing the HMO degradant sugars in the spent media^22^. Interestingly, the results revealed that *B*. *bifidum* JCM1254, belonging to the first type of HMO degraders, leaves some of the HMO degradant sugars unconsumed during growth, despite that the strain can theoretically assimilate all types of HMOs (Fig. 1)^23–26^. We found that the culture supernatant of early exponential phase of *B*. *bifidum* JCM1254 grown in the presence of HMOs contained significant amounts of lacto-*N*-biose I (LNB: Galβ1-3GlcNAc) and lactose (Lac: Galβ1-4Glc), which are produced from lacto-*N*-tetraose (LNT: Galβ1-3GlcNAcβ1-3Galβ1-4Glc, the most abundant HMO core structure) by lacto-*N*-biosidase (LnbB)^22^. In addition, galactose (Gal) was detected throughout the culture period, and remained unconsumed even at the end of stationary phase. LnbX-positive *B*. *longum* JCM1217 also transiently released Lac into the culture. Given these results, we hypothesised that the HMOs degradants may be symbiotically shared among different species of *Bifidobacterium* in the gut community, as LNB, Lac, and Gal serve as good carbon sources^16,22^. In particular, LNB selectively stimulates the growth of the four infant gut-associated bifidobacterial species among the different gut microbes^27^. LNB assimilation requires an ABC transporter (Galacto-*N*-biose (GNB)/lacto-*N*-biose I (LNB) transporter) and a cytoplasmic phosphorylase (GNB/LNB phosphorylase)^27,28^, both of which are essentially limited to *B*. *longum*, *B*. *breve*, *B*. *bifidum*, and *B*. *infants*, with a few exceptions (Figs 1 and S1)^27^. In 2013, Tannock *et al*. reported that the presence of *B*. *bifidum* in the gut microbiome is associated with an increased prevalence of bifidobacteria at the genus level in the stools of breast-fed babies^5^. Interestingly, the relationship was absent in the stools of formula-fed babies. These results imply cross-feeding of HMOs degradants produced by *B*. *bifidum* within the bifidobacterial community of the gut. The results also suggest that extracellular glycosidase-dependent bifidobacterial species may act in an altruistic manner, while oligosaccharide transporter-dependent bifidobacterial species appear more selfish.

In the current study, we examined this altruistic behaviour of *B*. *bifidum* using four strains isolated from infant faeces. The strains were first assayed for *in vitro* HMOs assimilation by analysing the sugars (HMOs degradants) present in the spent media. Then, *B*. *bifidum*-mediated cross-feeding of HMOs degradants to *B*. *longum* was demonstrated by simple co-culture experiments. In addition, we examined for ability of the four *B*. *bifidum* strains to promote the growth of other bifidobacterial species in assays using HMOs-supplemented faecal samples. The complete genome sequences of two *B*. *bifidum* strains were also determined. The results suggest a functional commonality among *B*. *bifidum* strains as cross-feeders of HMOs degradants, which could advance our knowledge of how bifidobacteria-rich microbiota are formed in the guts of breast-fed infants, and how we could intervene to shape the bifidus flora in the infant gut.

## Results

### Growth of four *B*. *bifidum* strains in the presence of HMOs

Four *B*. *bifidum* strains, all of which were isolated from infant stools, were cultivated in medium containing HMOs as the sole carbon source. Lac-containing medium was used for comparison. All four strains assimilated both HMOs and Lac (optical density at 600 nm (OD_600_) >0.5 within 15 h) (Fig. S2), and did not grow in the absence of a supplemented carbon source (OD_600_ < 0.05 after 20 h) (data not shown). Strains JCM1254, TMC3108, and MC3115 achieved higher cell biomass than strain JCM7004 in HMO-supplemented medium under the tested conditions.

### Complete genome sequencing of *B*. *bifidum* strains JCM7004 and TMC3115

**Figure 2:**
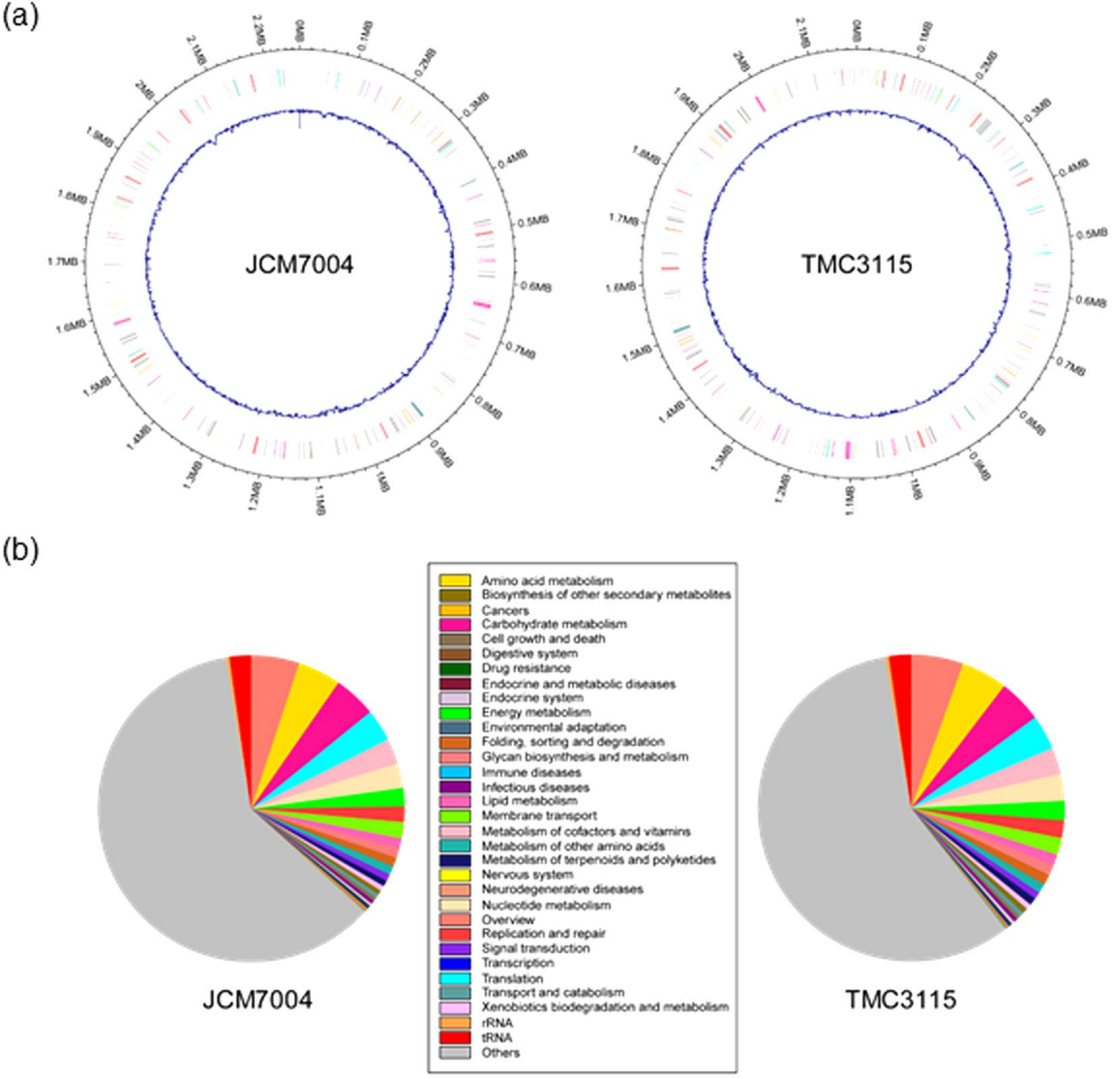
Schematic representation of the genomic structures (**a**) and gene functions (KEGG Orthology) (**b**) of *B*. *bifidum* strains JCM7004 and TMC3115. The genome structures were depicted using the R package “circlize”. Blue lines represent GC content. See also Tables S1 and S2.

The draft genome sequence of *B*. *bifidum* strain JCM1254 is available from the GenBank database (Whole Genome Shotgun Sequencing Project accession number BBBT00000000). In the present study, we determined the complete genome sequences of strains JCM7004 and TMC3115 (Fig. 2 and Tables S1 and S2). These two strains were chosen for analysis because they scavenge mucin-type glycans better than strains JCM1254 and TMC3108^26^ (unpublished results). The circular chromosome of strain JCM7004 was 2,261,666 bp in length, and was predicted to contain 2,106 coding sequences (CDS), 57 tRNA genes, and 3 rRNA operons. The chromosome of strain TMC3115 comprised 2,178,894 bp, and was predicted to contain 1,876 CDS, 53 tRNA genes, and 3 rRNA operons. The average GC contents of the genomes were 62.6% and 62.8%, respectively. Among the seven available *B*. *bifidum* genome sequences, the genome of strain JCM7004 was the largest, while that of strain TMC3115 was the smallest (Table S1). Kyoto Encyclopaedia of Genes and Genomes (KEGG) orthology analysis assigned 932 functions to the 2,106 CDS of strain JCM7004, and 905 functions to the 1,876 CDS of strain TMC3115 (Fig. 2 and Table S2). Among the annotated genes of both strains, more than 10% were predicted to be involved in carbohydrate metabolism. Database for Carbohydrate-Active Enzyme Annotation analysis^29^ revealed that the genomes of JCM7004 and TMC3115 contained 40 and 39 glycosidase hydrolase (GH)-family domains classified in the Carbohydrate-Active Enzyme database (http://www.cazy.org/)^30^, respectively. The abundance of GH-family domains, especially those dedicated to host glycan foraging, in the genomes of *B*. *bifidum* strains has been described previously^31^.

### Conservation of HMO-degrading enzymes among *B*. *bifidum* strains

Previous studies using strain JCM1254 have revealed that six extracellular enzymes, one transporter, and one intracellular phosphorylase play pivotal roles in assimilating HMOs in this species (Fig. 1). 1,2-α-l-Fucosidase (AfcA)^23^, 1,3-1,4-α-l-fucosidase (AfcB)^24^, LnbB^25^, β-1,4-galactosidase III (BbgIII), β-1,3-*N*-acetylglucosaminidase I (BbhI)^26^, and sialidase II^32^ are cell surface-anchored secretory enzymes that degrade all types of HMO structures into monosaccharides (Fuc, Gal, Glc, *N*-acetylglucosamine (GlcNAc), and Neu5Ac) and disaccharides (Lac and LNB) extracellularly. LNB is imported by the GNB/LNB transporter into the cells^22^, and is then phosphorolysed by GLNBP into galactose-1-phosphate and GlcNAc^33^ (Fig. 1), which enter the so-called bifido shunt glycolytic pathway^34^. Lac is either hydrolysed by BbgIII or imported by Lac permease^35^. AfcA, AfcB, LnbB, BbgIII, BbhI, the GNB/LNB transporter, and GLNBP show the highest preference for host-derived glycan structures. These seven enzymes constitute the minimum set required for complete digestion of neutral HMOs, which comprise >90% of the total HMOs^14^, by *B*. *bifidum* JCM1254 (Fig. 1).

Genome analysis revealed that both JCM7004 and TMC3115 contain all of the genes for the above-mentioned minimum set of HMO-degrading enzymes (Table 1). Strain TMC3108 was also found to have these genes, as revealed by direct sequencing of the polymerase chain reaction (PCR) products. Amino acid sequence identity was very high (97.3‒100%) among the respective seven enzymes from strains JCM1254, JCM7004, TMC3108, and TMC3115. Moreover, among the enzymes for which the structures have been solved (AfcA, AfcB, LnbB, the GNB/LNB transporter solute-binding protein (GL-BP), and GLNBP), the amino acid residues involved in substrate recognition and catalysis were completely conserved (Fig. S3a–e). The other five *B*. *bifidum* strains whose genome sequences have been reported (listed in Table S1) also contained genes coding for the complete set of enzymes, with amino acid sequence identities of >98.1%. One exception was the GL-BP of strain JCM1255^T^, where a frameshift mutation had been introduced^22^, which may account for the limited growth of the strain in HMOs-supplemented medium^17^. The highly conserved nature of the HMO-degrading enzymes in *B*. *bifidum* at the species level is in sharp contrast to findings from other infant gut-associated bifidobacterial species. In *B*. *longum*, *B*. *infantis*, and *B*. *breve*, the presence of genes encoding the HMO-degrading enzymes is strain dependent, except for the GNB/LNB transporter and GLNBP^6,7,15,19–21^ (Fig. S1).

**Table 1:** Conservation of HMO-degrading enzymes in *B*. *bifidum*.

Enzymes	JCM1254		JCM7004^a^	TMC3108^b^	TMC3115^a^
	Gene (GenBank ID)	(ref)	Amino acid sequence identity (%)^c^		
*Cell wall-anchored secretory enzymes*					
1,2-α-l-Fucosidase (AfcA)	*afcA* (AAQ72464)	^23^	98.5	98.7	99.1
1,3-1,4-α-l-Fucosidase (AfcB)	*afcB* (BAH80310)	^24^	97.6	97.3	97.4
Lacto-*N*-biosidase (LnbB)	*lnbB* (ABZ78855)	^25^	99.5	99.6	99.6
β-Galactosidase III (BbgIII)	*bbgIII* (BAI94821)	^26^	98.8	98.7	99.0
β-*N*-Acetylglucosaminidase I (BbhI)	*bbhI* (BAI94822)	^26^	99.7	99.5	99.7
*Transporter*					
Solute-binding protein (GL-BP) of the GNB/LNB transporter	*gltA* (AEP83683)	^22^	98.9	98.6	98.6
*Cytoplasmic enzyme*					
GNB/LNB phosphorylase (GLNBP)	*lnpA1* (BAD80752)	^33^	100	99.6	99.2

^a^Complete genome sequences were determined in this study.

^b^Sequences were determined by direct sequencing of PCR products. Accession numbers are indicated in the Methods section.

^c^Amino acid sequences were aligned using Clustal Omega^58^. The alignment and 3D structures are shown in Fig. S3. The structures of BglIII and BbhI have not yet been solved.

### HMOs consumption behaviour of the four *B*. *bifidum* strains

**Figure 3:**
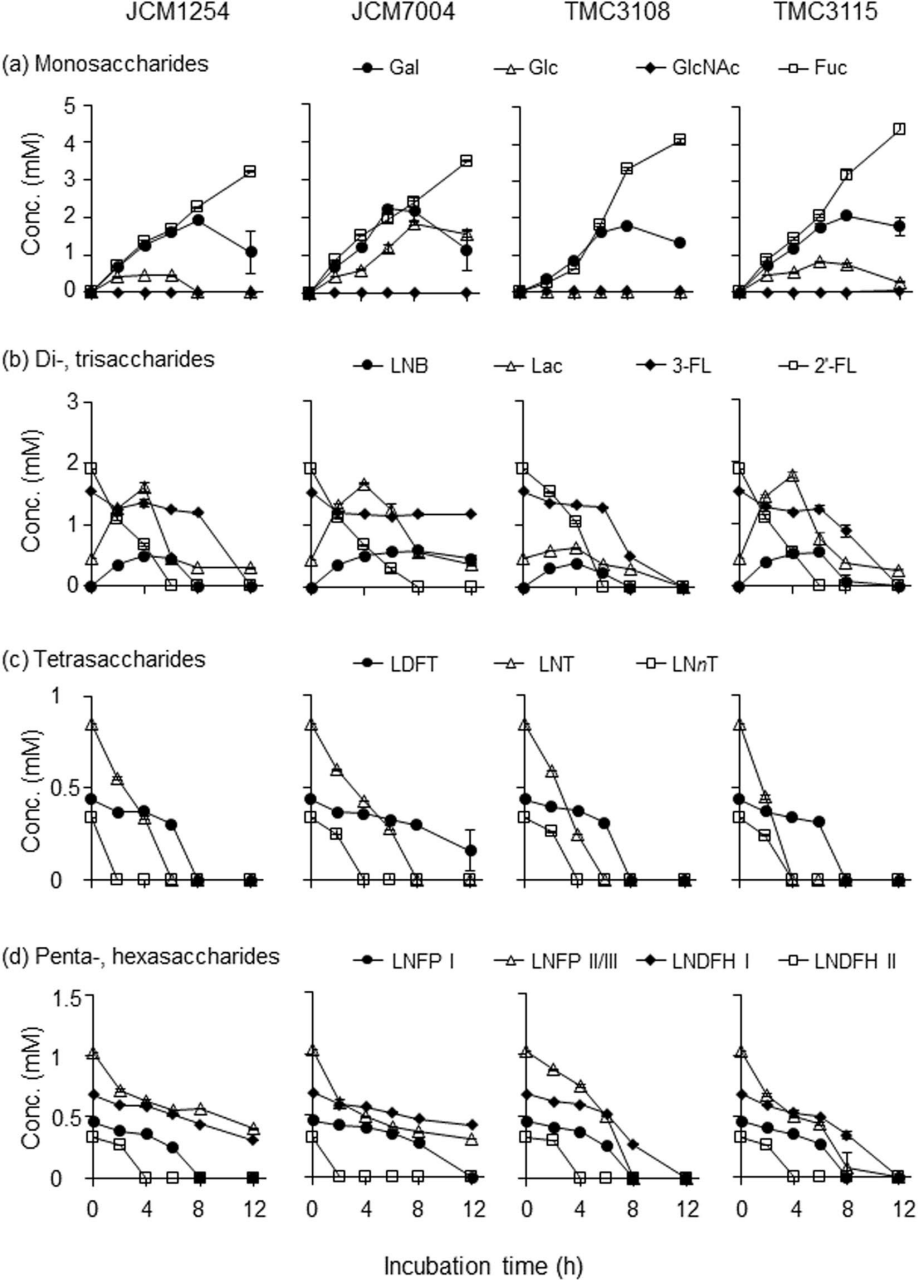
*In vitro* HMO degradation behaviour of four *B*. *bifidum* strains. Each strain was cultured in HMOs-containing basal media in triplicate, and samples were collected at the indicated time points (see Fig. S2). The sugars in the culture supernatants were labelled with 2-AA and analysed by HPLC, as described in the Methods section. Note that LNB was not accurately quantified due to its heat lability. Concentrations of (**a**) monosaccharides (Fuc, Gal, Glc, and GlcNAc), (**b**) di- and trisaccharides (2′-FL, 3-FL, Lac, and LNB), (**c**) tetrasaccharides (LNT, LN*n*T, and LDFT), and (**d**) penta- and hexasaccharides (LNFP I, LNFP II/III, LNDFH I, and LNDFH II) are shown. The data are means ± SD of the labelled sugars obtained from three separate cultures.

To verify the role of the seven HMO-degrading enzymes in *B*. *bifidum* during growth, we analysed the sugar composition and concentration in the spent media of the four strains cultured in medium supplemented with neutral HMOs. HMOs were purified from pooled milk of 14 Japanese mothers. The samples were collected at the indicated time points (Fig. S2) and subjected to a normal-phase high performance liquid chromatography (HPLC) following 2-aminoanthranilic acid (2-AA)-labelling of mono- and oligosaccharides (Figs 3 and S4 and Table S3). The results revealed that HMO consumption behaviours were very similar among the four strains, reflecting the conserved nature of the enzymes. The HMO degradation patterns were also similar to our previous observations^22^. The degradation of LNT and 2′-fucosyllactose (2′-FL), which are the major components of HMOs, began prior to cells entering exponential phase, and the majority of these two substrates was degraded by early exponential phase, indicating the rapid action of LnbB and AfcA, respectively^23–25,36^. As a result, transient increases in the concentrations of LNB (at least 0.3 mM, see the Methods section) and Lac (up to 1.9 mM) were observed. Gal was liberated from lacto-*N*-*neo*tetraose (LN*n*T: Galβ1-4GlcNAcβ1-3Galβ1-4Glc) and Lac by BbgIII, and accumulated in the spent medium throughout the growth period. Glc concentrations remained low during growth except for strain JCM7004. The impaired Glc consumption ability of JCM7004 might account for lower cell biomass formation of the strain than the other three strains during growth (Fig. S2). GlcNAc was not detected throughout the growth period. α-(1 → 3/4)-Fucosyl linkages present in 3-fucosyllactose (3-FL), lactodifucotetraose (LDFT), lacto-*N*-fucopentaose II/III (LNFP II/III), and lacto-*N*-difucohexaose I/II(LNDFH I/II) were hydrolysed by AfcB. The slow degradation of 3-FL and LNDFH I can be explained by the low catalytic activity of AfcB compared with AfcA^37^. These results revealed that the release of HMOs degradants (LNB, Lac, Gal, and Fuc) into culture supernatants is part of the intrinsic nature of *B*. *bifidum* species, the extracellular glycosidase-dependent HMOs consumers.

### *B*. *bifidum*-mediated cross-feeding of HMOs degradants to *B*. *longum*

**Figure 4:**
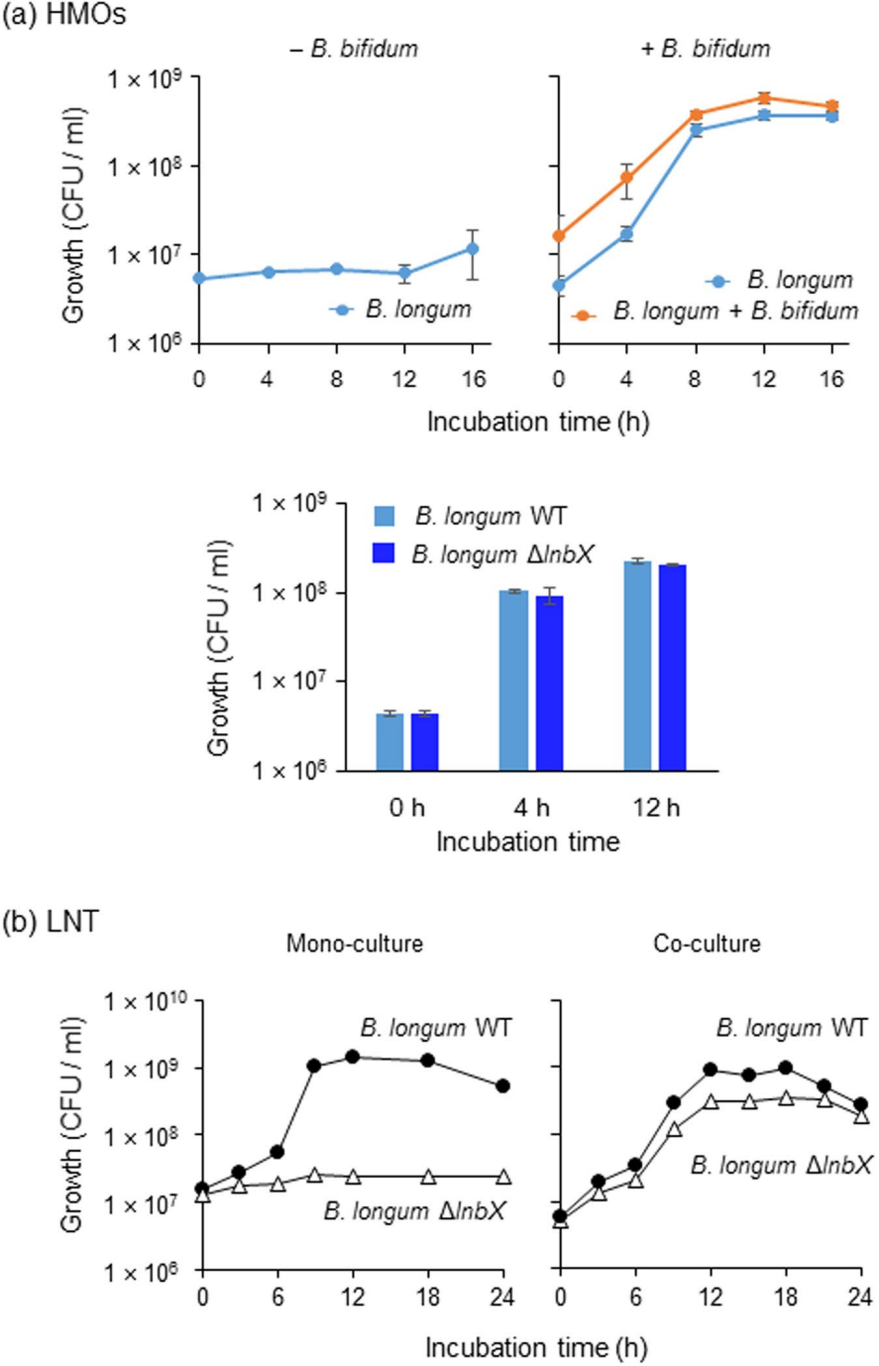
Inter-species cross-feeding of HMO degradants among bifidobacteria (**a**). WT strain of *B*. *longum* 105-A carrying pBFS38 (Cam^R^) was grown in basal medium supplemented with 1% HMOs in the absence (upper left panel) and presence (upper right panel) of *B*. *bifidum* JCM1254. Samples were taken at the indicated time points, and CFUs were determined by spreading the diluted cultures on Cam-containing (for *B*. *longum* cells) and not containing (for *B*. *longum* + *B*. *bifidum* cells) agar plates. For competition assay in the presence of *B*. *bifidum* JCM1254, WT and its isogenic Δ*lnbX* strains of *B*. *longum* 105-A were transformed with pBFS38 (Cam^R^) and pBFO2 (Sp^R^), respectively, to distinguish between them (lower panel). (**b**) Intra-species cross-feeding of LNT degradants. WT strain of *B*. *longum* 105-A carrying pBFS38 (Cam^R^) and its Δ*lnbX* variant carrying pBFO2 (Sp^R^) were cultured in basal medium containing 1% LNT as a carbon source, separately (mono-culture) or in combination (co-culture). Growth was monitored by calculation of CFUs. Identical results were obtained when the marker genes were exchanged between the strains. The data are means ± SD of three independents experiments (**a**,**b**).

To examine the possibility of the symbiotic sharing of HMO degradants among bifidobacteria, we attempted to disrupt the *afcA* (1,2-α-l-fucosidase) or *lnbB* (lacto-*N*-biosidase) genes in the *B*. *bifidum* strains. However, all attempts were unsuccessful. Therefore, we performed simple *in vitro* co-culturing of *B*. *longum* 105-A and *B*. *bifidum* JCM1254 in HMOs-supplemented medium (Fig. 4a upper panel). Whereas wild-type (WT) strain of *B*. *longum* 105-A (LnbX^+^) showed very limited growth on HMOs in mono-culture, it grew well when co-cultured with *B*. *bifidum*, suggesting cross-feeding of HMOs degradants at the inter-species level. Growth competition experiments showed that the *lnbX* gene is dispensable for *B*. *longum* to utilize HMOs degradants produced by *B*. *bifidum* (Fig. 4a lower panel). However, the gene was found to be indispensable for the strain when grown in LNT as a sole carbon source. The Δ*lnbX* derivative of *B*. *longum* 105-A was incapable of assimilating LNT in mono-culture, while it grew well in the presence of WT *B*. *longum* 105-A (LnbX^+^) (Fig. 4b), implicating that WT LnbX^+^ cells can feed Δ*lnbX* cells with LNT degradants (Lac and/or LNB). Such intra-species level cross-feeding may also occur in gut microbial community.

### Effects of *B*. *bifidum* supplementation on the growth of other *Bifidobacterium* species in faecal cultures containing HMOs

**Figure 5:**
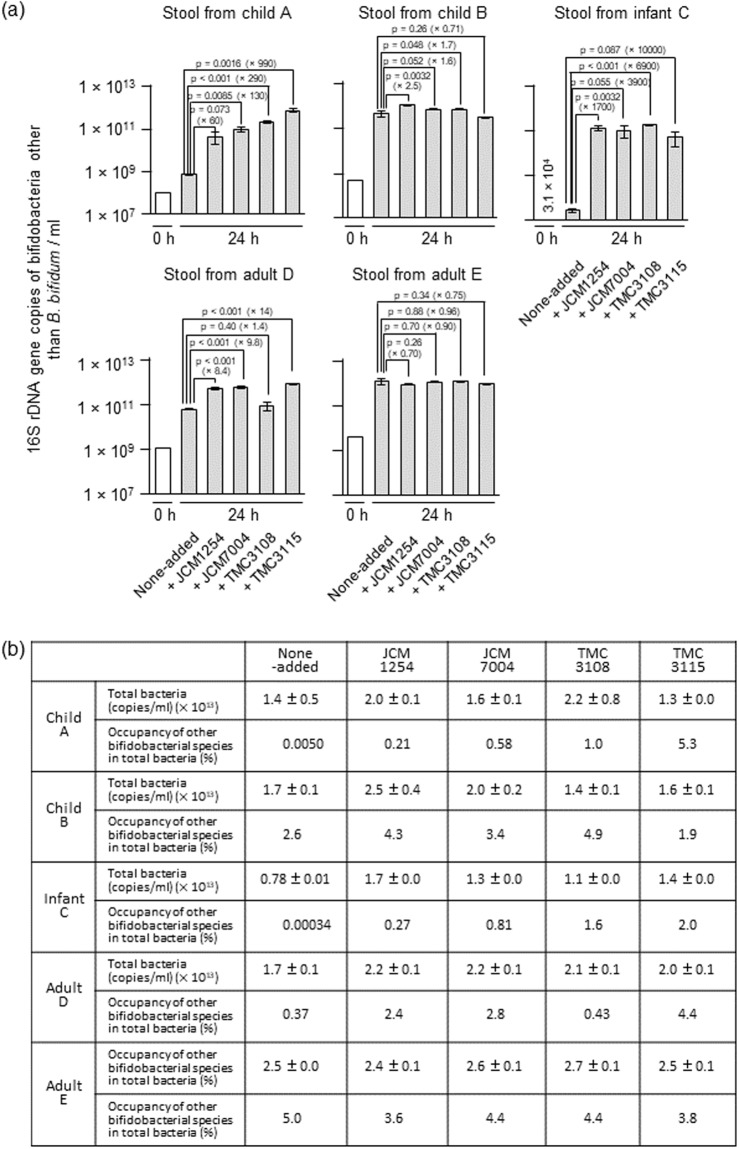
Addition of *B*. *bifidum* to faecal suspensions incubated in the presence of HMOs enriches the *Bifidobacterium* population (species other than *B*. *bifidum*) in the culture. (**a**) Stool suspensions from two children (4-year-old female and 5-year-old male), one infant (caesarean delivered 4-month-old preweaning female), and two adults (30-year-old male and 39-year-old male) were cultured in basal medium containing 1% HMOs with and without the addition of four *B*. *bifidum* strains for 24 h. The abundance of 16S rRNA gene sequences corresponding to *Bifidobacterium* species other than *B*. *bifidum* were determined by qPCR at 0 h (white bars) and 24 h (grey bars) post-inoculation, as described in the Methods section. The data are means ± SD of three independents assays. Dunnett’s test was used to examine the statistical significance. (**b**) Prevalence of *Bifidobacterium* species other than *B*. *bifidum* in the culture. The total bacterial population was determined as described in the Methods section. Prevalence was calculated by dividing bifidobacterial 16S rRNA gene counts (except for *B*. *bifidum*) by total bacterial 16S rRNA gene counts. See also Figs S5–S8 and Tables S4 and S5.

To determine whether HMOs degradants produced by *B*. *bifidum* can be shared by other bifidobacterial species in complex ecosystem, we performed *in vitro* co-culture experiments using stool samples collected from one infant, two young children, and two adults (Table S4). The initial abundance of 16S rRNA gene copies attributed to members of the genus *Bifidobacterium* in stool suspensions from child A, child B, infant C, adult D, and adult E was 1.0 × 10^8^, 4.7 × 10^8^, 3.1 × 10^4^, 1.1 × 10^9^, and 3.9 × 10^9^ copies/mL, respectively, at 0 h (Fig. 5a). The 16S rRNA gene copies specifically attributed to *B*. *bifidum* was below the detection limit of our quantitative PCR (qPCR) system (<4.0 × 10^4^ copies/mL) in all samples except for adult D sample (Fig. S5). The stool suspensions were incubated for 24 h in medium supplemented with 1% HMOs, either in the presence or absence of each of the four *B*. *bifidum* strains. Addition of *B*. *bifidum* considerably stimulated the growth of other *Bifidobacterium* species in all cases, except for adult E samples and for TMC3115-added child B sample. Statistical significances were detected in 10 out of 15 cases for which the stimulatory effects of *B*. *bifidum* were observed (*p* < 0.05, Dunnett’s test) (Fig. 5a). In stool cultures from child A, the observed growth promotive effect was 60-, 130-, 290-, and 990-fold for strains JCM1254, JCM7004, TMC3108, and TMC3115, respectively, compared with the control culture without supplementation of *B*. *bifidum*. For stool cultures from child B, we observed 2.5-, 1.6-, and 1.7-fold increases in the number of 16S rRNA copies attributed to other bifidobacterial species in the presence of strains JCM1254, JCM7004, and TMC3108, respectively, compared with the control. In the case of stool sample obtained from infant C, the growth of other bifidobacterial species was stimulated by 1,700- to 10,000-fold by adding *B*. *bifidum* strains, while 1.4- to 14-fold stimulation was observed for the sample from adult D, compared with none-added control. Consequently, the prevalence of bifidobacterial species other than *B*. *bifidum* in total bacteria was increased when *B*. *bifidum* strains were added to the faecal samples in all cases, except for the child B stool sample supplemented with TMC3115 and for the adult E samples (Fig. 5b). Particularly, drastic increases of the prevalence of other bifidobacterial species in total bacteria were observed in faecal cultures from the child A and infant C samples. It should be noted that although the *B*. *bifidum* 16S rRNA gene was not detected in the control cultures from stool samples from child A and infant C after 24 h, it was detected in the non-added control cultures of stool samples from child B, adult D, and adult E after 24 h incubation (1.8 × 10^6^, 3.1 × 10^9^, and 1.5 × 10^7^ copies/mL, respectively) (Fig. S5a). The results indicate the latter three faecal samples contained *B*. *bifidum* cells endogenously when collected. The culture supernatants were also analysed by thin-layer chromatography (TLC) (Fig. S6a). In the control cultures of stool samples from child A and infant C, the spots corresponding to 2′-FL, LNFP I, LNDFH I, and longer HMOs appeared to be undigested even after cultivation for 24 h. However, when the stool samples were co-cultivated with each of the four *B*. *bifidum* strains, these oligosaccharides were almost completely consumed. In stool sample B, a spot corresponding to LNDFH I was detected in the control culture, but disappeared when the four *B*. *bifidum* strains were added. In the supernatant of cultures of adult D and adult E samples, no obvious spots were detected in the TLC analysis both for the control and *B*. *bifidum*-added cultures after 24 h incubation.

In contrast to the results obtained from HMOs-containing cultures, when the same experiments were conducted using Glc as the carbon source, the growth stimulatory effects of *B*. *bifidum* were not evident (Fig. S7a). Addition of *B*. *bifidum* JCM1254 and TMC3108 to the stool suspensions from child A and infant C, respectively, increased the 16S rRNA gene copy numbers of other *Bifidobacterium* species by 5.6- and 9.3-fold; however, in the other cultures, the copy numbers were unchanged or decreased. Reflecting these results, bifidobacterial populations other than *B*. *bifidum* in total bacteria were also unchanged or decreased considerably compared with those of the control cultures that were not supplemented with *B*. *bifidum* (Fig. S7b). Regardless of the carbon source used (HMOs or Glc), the supplemented *B*. *bifidum* strains grew well in the faecal cultures (Fig. S5). The final pHs of the broths were neutral (6.4‒7.7) when the faecal suspensions were cultured in the presence of HMOs, while they dropped to 5.1‒6.1 when incubated with Glc (Table S5). Taken together, these results strongly suggest that HMO degradants produced by *B*. *bifidum* are used to feed not only themselves but also other bifidobacterial species, thereby increasing the prevalence of bifidobacteria in faecal cultures. The growth stimulatory effect was found to be less effective when the collected faecal sample intrinsically contains *B*. *bifidum* cells already.

## Discussion

The intestinal microbial composition is influenced not only by host-microbe interactions, but also by microbe-microbe interactions in the gut. Competition between bacterial species in the gut is a well-known example of microbe-microbe interactions, and can help prevent colonization by invading pathogenic bacteria^38^. However, recent studies have revealed complex microbe-microbe interactions involving cross-feeding among different microbes. Cross-feeding is thought to occur both bidirectionally and unidirectionally. Bidirectional feeding is observed between *Bacteroides ovatus* and *Bacteroides vulgatus*, which is mediated through inulin^39^, and between *Akkermansia muciniphila* and *Eubacterium hallii*, which is mediated through *O*-glycan degradants derived from mucin and pseudovitamin B12^40^. Unidirectional feeding is found between *A*. *muciniphila* and *Anaerostipes caccae*, in which acetate produced by *A*. *muciniphila* stimulates the growth of *A*. *caccae*^40^, and between *Bifidobacterium adolescentis* and *Faecalibacterium prausnitzii*, whereby acetate that is required for the growth of *F*. *prausnitzii* is provided by *B*. *adolescentis*^41^. Apparently, intra- and inter-genus feeding of degradants and metabolites among bacteria increases gut microbiota diversity, which is thought to be important for shaping a flexible, healthy gut environment^42^.

Over the last decade, several groups, including our own, have focused on elucidating the mechanism of HMOs degradation by bifidobacteria. These studies revealed that HMOs assimilation is essentially limited to infant gut-associated bifidobacterial species represented by *B*. *infantis*, *B*. *longum*, *B*. *breve*, and *B*. *bifidum*. Interestingly, although the four species belong to the same genus, they have evolved different strategies to degrade HMOs (i.e., the extracellular glycosidase-dependent one adopted by *B*. *bifidum* and LnbX-positive *B*. *longum*, or the transporter-dependent one observed in *B*. *breve*, *B*. *infantis*, and LnbX-negative *B*. *longum*), resulting in varied HMO consumption behaviours. LoCascio *et al*. found that *B*. *infantis* strains that grow in the presence of HMOs share a 40-kb gene cluster encoding ABC transporters and intracellular exoglycosidases that act on HMOs, allowing *B*. *infantis* to consume most of HMOs^43^. *B*. *bifidum* is also an avid consumer of HMOs, and in the present study, we demonstrated that this phenotype can be attributed to the conserved presence of HMOs-degrading enzymes in this species. In contrast to *B*. *infantis* and *B*. *bifidum*, *B*. *breve* and *B*. *longum* are fastidious about HMOs consumption. The two species commonly consume LNT, but assimilation of the other HMOs is variable and strain-dependent^19,20^. Some *B*. *breve* strains can degrade LN*n*T, 2′-FL, and/or 3-FL^6,18^, and some *B*. *longum* strains can consume 2′-FL and LNFP I in addition to LNT. Although 2′-FL, 3-FL, and LNFP I constitute major HMOs (Table S3), breast-fed infant *B*. *breve* and *B*. *longum* faecal isolates frequently lack the genes required to assimilate these HMO molecules^19^. Consequently, at the species level, the ability of *B*. *breve* and *B*. *longum* strains to assimilate HMOs is limited compared with *B*. *infantis* and *B*. *bifidum*. Nonetheless, interestingly, infant gut microbiota is frequently dominated by *B*. *breve* and/or *B*. *longum*^6,15^. Thus, the formation of a bifidobacteria-rich microbiota in the breast-fed infant gut is not simply explained by HMOs assimilation phenotypes.

Our previous findings showed that *B*. *bifidum* JCM1254 leaves some HMOs degradants unconsumed during growth, while a report by Tannock *et al*. described the positive relationship between occurrence of *B*. *bifidum* and the abundance of *Bifidobacterium* at the genus level in the faeces of breast-fed infants^5^. These findings led us to consider the possibility of *B*. *bifidum*-mediated cross-feeding of HMOs degradants to other bifidobacterial species. First, we showed that all four *B*. *bifidum* strains examined release HMOs degradants during cultivation, and then demonstrated that inter- and intra-species cross-feeding of HMOs degradants among bifidobacteria. *B*. *longum* proliferated in HMOs-containing broth only when co-cultured with *B*. *bifidum* (Fig. 4). Whereas lacto-*N*-biosidase-deficient *B*. *longum* strain did not assimilate LNT, it grew well on LNT when co-cultivated with its parental WT *B*. *longum* strain. Given the results, we extended our hypothesis to more complex ecosystem, e.g. faecal samples. Our current findings show that, when added to faecal cultures, the four *B*. *bifidum* strains considerably stimulate the growth of other bifidobacterial species (except for the adult E samples). In addition, HMOs that remained unconsumed in the control cultures of stool samples from child A, child B, and infant C disappeared when the faecal samples were co-cultivated with *B*. *bifidum*. Growth promotive effects were evident in HMOs-containing cultures, but not apparently observed in Glc-containing culture (Fig. 5 vs. Fig. S7). It is interesting to note that *B*. *bifidum* was detected in the non-added control cultures of faecal samples B, D, and E after 24 h of cultivation in the presence of HMOs, but was not detected in the control cultures of faecal samples A and C. Considering that the growth-promoting effects of *B*. *bifidum* were more evident in faecal samples A and C than in faecal samples B, D, and E, endogenously present *B*. *bifidum* in the collected stool samples might assist the growth of other bifidobacterial species in the non-added control cultures of faecal samples B, D, and E, which slightly (for B and D) or completely (for E) masked the stimulatory effects of exogenously supplemented *B*. *bifidum* strains (Figs 5 and S5). It should be mentioned, in terms of intervention, that bifidobacterial population and occupancy was greatly increased in HMOs-supplemented stool sample obtained from infant C, who was a caesarean-delivered preweaning infant.

Finally, we examined the effect of α-fucosidase inhibitor deoxyfuconojirimycin (DFJ)^37^ on the cross-feeding of HMOs degradants among bifidobacteria. Addition of DFJ to the HMOs-supplemented faecal cultures of child A completely abolished the growth stimulatory effects of the all *B*. *bifidum* strains (Fig. S8). The results could be caused by DFJ-mediated inhibition of hydrolysis of fucosylated HMOs, because the TLC analysis of the spent media revealed that 2′-FL, LNFP I, and LNDFH I remain unconsumed after 24 h incubation (Fig. S6b). Thus, extracellular glycosidase-dependent HMO-consumers may at least be involved in unidirectional cross-feeding of other microbes. These results strongly suggest altruistic behaviour by *B*. *bifidum* (and probably LnbX^+^-*B*. *longum*) to establish a bifidobacteria-rich microbiota in the gut of breast-fed infants, although we cannot rule out the possibility that an unidentified metabolite produced from HMOs by *B*. *bifidum* stimulates the growth of other bifidobacterial species. The altruistic behaviour of *B*. *bifidum* may also occur in adult intestines. Egan *et al*. described possible cross-feeding of mucin degradants produced by *B*. *bifidum* to *B*. *breve*^44,45^. Mucin is a highly glycosylated protein that is abundantly expressed in the human large intestine, and *B*. *bifidum* possesses cell wall-anchored secretory glycosidases to degrade the sugar chains^16,46,47^. Interestingly, most of the mucin-degrading glycosidases are commonly used for degradation of HMOs^16^, reflecting the similarities in the glycoside linkages between mucin glycans and HMOs.

In summary, our findings reveal conservation in HMO-degrading enzymes and HMO consumption behaviour among *B*. *bifidum* strains, and suggest that *B*. *bifidum* could serve as a key player to establish a bifidobacteria-rich microbiota in the breast-fed infant gut, by providing HMOs degradants. These results enhance our understanding of how the bifidus flora is formed during infancy, and provide insight into how we could fortify prebiotic and probiotic foods, including infant formula, to enhance the bifidobacterial population in the infant gut.

## Methods

### Chemicals

2′-FL, 3-FL, LN*n*T, LNFP I, and LNDFH II were purchased from Dextra Laboratory (Reading, UK). LDFT and LNT were obtained from IsoSep (Tullinge, Sweden), or as gifts from Glycom A/S (Denmark). LNFP II and LDFH I were purchased from Carbosynth (Berkshire, UK). DFJ was obtained from Sigma-Aldrich (MO, USA). Isomaltopentaose was from Seikagaku Kogyo (Tokyo, Japan), while LNB was synthesized as previously described^48^. 2-AA and sodium cyanoborohydride were obtained from Nacalai tesque (Kyoto, Japan). All other reagents were of analytical grade.

### Bacterial strains and culture conditions

Bacteria used in this study included *B*. *bifidum* strains JCM1254, JCM7004, TMC3108, and TMC3115, along with *B*. *longum* 105-A and its lacto-*N*-biosidase gene disruptant Δ*lnbX*^7,49^. Strains JCM1254 and JCM7004 were obtained from the Japan Collection of Microorganisms (JCM; RIKEN Bioresource Center, Japan), while strains TMC3108 and TMC3115 were isolated previously from infant stools^47,50^. *B*. *longum* 105-A was obtained from Dr. Yasunobu Kano^49^. All strains were routinely grown in GAM broth (Nissui Pharmaceutical, Tokyo, Japan) under anaerobic conditions using the AnaeroPack system (Mitsubishi Gas Chemical Co., Tokyo, Japan). When examining growth in medium supplemented with HMOs, basal medium (0.2% yeast extract, 1.0% peptone, 0.5% sodium acetate, 0.2% diammonium citrate, 0.02% magnesium sulphate, 0.2% dipotassium hydrogen phosphate, and 0.1% cysteine hydrochloride) was used. The medium was supplemented with 4% reducing reagent (2% cysteine hydrochloride and 11% sodium carbonate) and 1% sugar prior to inoculation. Three separate broths were inoculated with overnight culture of each strain and incubated anaerobically at 37 °C. Growth was monitored by measuring the OD_600_, or by determining CFU at the indicated time points. The data are expressed as means ± standard deviation (SD).

### Whole genome sequencing

Genomic DNA was extracted from *B*. *bifidum* strains JCM7004 and TMC3115 using a Wizard Genomic DNA Purification kit (Promega, WI), and was sequenced with ~540-fold and ~260-fold coverage, respectively, using a PacBio RS II sequencer (Pacific Biosciences, CA). The sequences were assembled using SMRT Analysis software v2.3.0. Additional sequencing was carried out using a HiSeq. 2500 system (Illumina, San Diego, CA) to fill the gaps. Sequencing and analysis were performed by the Dragon Genomic Center at TaKaRa Bio (Shiga, Japan), Eurofins Genomics (Tokyo, Japan), and Beijing Genomics Institute (Guangdong, China). Glimmer3^51^ was used to predict open reading frames, and gene annotations were assigned by KAAS^52^, based on the KEGG^53^ database.

### Direct sequencing of PCR products

The genes coding for the HMO-degrading enzymes of *B*. *bifidum* TMC3108 were amplified in high-fidelity PCR using PrimeSTAR Max (TaKaRa Bio) and genomic DNA as a template. The primers used for amplification are listed in Table S6. The amplified fragments were purified, and then directly sequenced using a primer walking method.

### Preparation of HMOs from human milk

HMOs were purified from human milk as described previously^22^. Milk samples were collected at Nagao Midwife Clinics (Kyoto, Japan) from 14 healthy Japanese mothers who had not taken any antibiotics for 1 month prior to collection. Informed consent was obtained from all mothers.

### Sugar concentration analysis

Mono- and oligosaccharides in the culture supernatants were quantified by HPLC analysis following fluorescence-labelling of sugars. Aliquots (50 μL) of the culture incubated in the presence of HMOs were collected at the indicated time points, clarified by centrifugation, and stored at −20 °C until use. For the HPLC analysis, the supernatants were thawed and immediately mixed with 30 μL of water and 20 μL of 0.1% isomaltopentaose (internal standard). The sugars in the samples were then labelled with 2-AA, as described previously^22^. It should be noted that LNB is rapidly decomposed during heat treatment, and thus the concentrations shown in this paper correspond to less than half of its original concentrations in the medium. HPLC was carried out using a Waters e2695 separation module (Waters, Milford, MA) equipped with a TSKgel Amide-80 HR column (4.6 × 250 mm, φ = 5 μm) (Tosoh, Tokyo, Japan) at 65 °C. The column was equilibrated with 85% solvent A (acetonitrile)/15% solvent B (100 mM ammonium formate buffer, pH 4.3). The elution was performed using a linear increase of solvent B (from 15% to 40%) over 90 min at a flow rate of 1 mL/min. The labelled sugars were detected at excitation and emission wavelengths of 330 and 420 nm, respectively, using a Waters 2475 Fluorescence Detector. The sugar concentrations were determined based on the standard curves generated using similarly labelled standard sugars. The data were normalised using the internal standard and expressed as means ± SD of the labelled sugars obtained from three separate cultures.

### *In vitro* co-culture of *B*. *longum* with *B*. *bifidum*

WT strain of *B*. *longum* 105-A with a plasmid carrying the chloramphenicol resistance gene (pBFS38)^54^ was cultured in basal medium supplemented with 1% HMOs as a carbon source in the presence and absence of *B*. *bifidum* JCM1254. During incubation, aliquots were collected, serially diluted, and spread on GAM agar plates supplemented with and without 2.5 μg/mL of chloramphenicol. Colonies appearing on the antibiotic-containing plates were attributed to *B*. *longum* cells, while those formed on antibiotic-free plates were assumed to represent the sum of *B*. *longum* and *B*. *bifidum* cells. When the Δ*lnbX* derivative of *B*. *longum* 105-A was competed against its parental WT strain (with pBFO2 carrying the spectinomycin resistance gene^55^) for the growth on HMOs in the presence of *B*. *bifidum*, pBFS38 was introduced into Δ*lnbX* strain to distinguish between them. WT and Δ*lnbX* derivative were also cultured separately or in combination in basal medium supplemented with 1% LNT as a carbon source. Different antibiotic resistance genes were introduced into the two strains by plasmids (pBFO2 or pBFS38) to distinguish between them. During incubation, aliquots were collected, serially diluted, and spread on GAM agar plates containing the respective antibiotics to determine the CFUs of each strain. Spectinomycin and chloramphenicol were used at the final concentrations of 30 μg/mL and 2.5 μg/mL, respectively.

### Faecal culture

Stool samples were collected from one healthy Japanese infant (infant-C, caesarean delivered 4-month-old preweaning female) and two healthy Japanese children (child A, 4-year-old female; child B, 5-year-old male) with their mothers’ consent and from two healthy Japanese adults (adult D, 30-year-old male; adult E, 39-year-old male) with their consent. The sample information is shown in Table S4. The samples were immediately stored under anaerobic conditions, and then transferred to the laboratory. The stools were suspended in 20% glycerol in an anaerobic chamber (InvivO_2_ 400; Baker Ruskinn, Bridgend, UK), and then frozen at −80 °C until use. Thawed stool samples A−E were used to inoculate 1 mL of basal medium containing 1% HMOs or Glc as a carbon source. At 0 h post-inoculation, the faecal suspensions from child A, child B, infant C, adult D, and adult E samples contained 16S rRNA gene copies attributed to total bacteria at the concentrations of 7.9 × 10^8^, 3.2 × 10^9^, 4.4 × 10^9^, 1.7 × 10^10^, and 1.7 × 10^10^ copies/mL, respectively. When needed, DFJ was added to the culture at a final concentration of 500 μM. Following further incubation for 24 h, the abundance of *B*. *bifidum* (species level) and *Bifidobacterium* (genus level) in each sample was examined by qPCR analysis of the 16S rRNA gene. In brief, each culture was centrifuged, and the precipitate was resuspended in 1 mL of TE buffer (10 mM Tris-HCl containing 1 mM EDTA, pH 8.0). The suspension was transferred to a 2-mL plastic tube containing a stainless steel bead (φ = 5.0 mm) and zirconia beads (φ = 0.1 mm, equivalent to 100 μL volume) before being shaken vigorously at 1,500 rpm for 10 min using a Shake Master Neo (Bio Medical Science, Tokyo, Japan). Total DNA was then extracted using a standard phenol-chloroform method from the lysate, and examined using the SYBR Green system (TaKaRa Bio). The data are means ± SD of three independent experiments. The prevalence of bifidobacteria was calculated by dividing bifidobacterial 16S rRNA gene counts (except for *B*. *bifidum*) by total bacterial 16S rRNA gene counts. The primers used are listed in Table S6. Spent media were used for measurement of the pH values with micro pH meter (HORIBA, Kyoto, Japan) and for TLC analysis with a silica gel 60 aluminium sheet (Merck, Darmstadt, Germany). The plate was developed in a solvent system consisting of 1-buthanol:acetic acid:water (2:1:1). Sugars were visualized as described previously^56^.

### Graphics

The genomic structure was schematically drawn using the R package “circlize” library^57^. Protein structure images were prepared using PyMOL (Schrödinger, Inc., NY). The amino acid sequences of HMO-degrading enzymes were aligned using Clustal Omega^58^, and the alignment was shown using BoxShade 3.2.1 (http://www.ch.embnet.org/software/BOX_form.html).

### Statistical analysis

Statistical analyses were performed using BellCurve version 2.00 (SSRI, Tokyo, Japan) and Excel 2013 (Microsoft) software. Dunnett’s test was used to examine the statistical significance, where p values of less than 0.05 were regarded as statistically significant.

### Ethical consideration

This study was reviewed and approved by the Ethics Committees of Kyoto University (R0046) and the University of Shiga Prefecture (71-3), and was conducted in accordance with the Declaration of Helsinki.

## Electronic supplementary material

Supplementary information

## Data Availability

The complete genomic sequences of *B*. *bifidum* JCM7004 and TMC3115 are available from the DDBJ under the accession numbers AP018131 and AP018132, respectively. The nucleotide sequences of the *afcA*, *afcB*, *lnbB*, *gltA*, *lnpA1*, *bbgIII*, and *bbhI* genes from *B*. *bifidum* TMC3108 were deposited in the DDBJ under accession numbers LC229083, LC229084, LC229085, LC229086, LC229087, LC229088, and LC229089, respectively.
