# Supplementary material for: Sharing of human milk oligosaccharides degradants within bifidobacterial communities in faecal cultures supplemented with Bifidobacterium bifidum
Source: Sci Rep. 2018 Sep 18;8:13958. doi: 10.1038/s41598-018-32080-3 (PMC6143587; doi:10.1038/s41598-018-32080-3)
Supplement: Supplementary file 1 — Supplementary information [file 41598_2018_32080_MOESM1_ESM.pdf]

Supplementary file

for

Sharing of human milk oligosaccharides degradants within bifidobacterial communities in faecal cultures supplemented with *Bifidobacterium bifidum*

Aina Gotoh,[1] Toshihiko Katoh,[1] Mikiyasu Sakanaka, [2] Yiwei Ling,[3] Chihaya Yamada,[1] Sadaki Asakuma,[4] Tadasu Urashima,[5] Yusuke Tomabechi,[2] Ayako Katayama-Ikegami,[2] Shin Kurihara,[2] Kenji Yamamoto,[2] Gaku Harata,[6] Fang He,[6] Junko Hirose,[7] Motomitsu Kitaoka,[8] Shujiro Okuda,[3]* and Takane Katayama[1,2]*

[1]Graduate School of Biostudies, Kyoto University, Sakyo-ku, Kyoto 606-8502, Japan
[2]Faculty of Bioresources and Environmental Sciences, Ishikawa Prefectural University, Nonoichi, Ishikawa 921-8836, Japan
[3]Graduate School of Medical and Dental Sciences, Niigata University, Chuo-ku, Niigata, 951-8510, Japan
[4]Hokkaido Agricultural Research Center, National Agriculture and Food Research Organization, Sapporo, Hokkaido 062-8555, Japan
[5]Obihiro University of Agriculture and Veterinary Medicine, Obihiro, Hokkaido 080-8555, Japan
[6]Takanashi Milk Products Co., Ltd., Yokohama, Kanagawa, 241-0023, Japan
[7]School of Human Cultures, The University of Shiga Prefecture, Hikone, Shiga 522-8533, Japan
[8]Food Research Institute, National Agriculture and Food Research Organization, Tsukuba, Ibaraki 305-8642, Japan

*Corresponding authors. Takane Katayama (takane@lif.kyoto-u.ac.jp) and Shujiro Okuda (okd@med.niigata-u.ac.jp)

Supplementary Table S1. Genome information for completely sequenced *B. bifidum* strains

Strain	length (bp)	CDS	tRNA	rRNA operons	GC content (%)	Glycoside hydrolases	Accession number or reference
JCM7004	2,261,666	2,106	57	3	62.6	40[a]	AP018131 (This study)
TMC3115	2,178,894	1,876	53	3	62.8	39[a]	AP018132 (This study)
JCM1255	2,211,039	1,831	53	2	62.7	49[b]	(1)
PRL2010	2,214,656	1,706	52	3	62.7	44[b]	(2)
S17	2,186,882	1,782	53	3	62.8	40[b]	(3)
BGN4	2,223,664	1,835	52	3	62.7	42[b]	(4)
BF3	2,210,370	1,696	52	3	62.6	45[b]	NZ_CP010412

See also Fig. 2.
[a]Number of glycoside hydrolases was predicted by dbCAN analysis (http://csbl.bmb.uga.edu/dbCAN/).
[b]Number of glycoside hydrolases was predicted based on analysis of the CAZy database (http://www.cazy.org/bB.html).

Supplementary Table S2. Functional assignments of the genes from *B. bifidum* strains JCM7004 and TMC3115, as predicted by KEGG Orthology Analysis.

KEGG Orthology	JCM7004	TMC3115
Metabolism/Amino acid metabolism	116	112
Metabolism/Biosynthesis of other secondary metabolites	12	13
Human diseases/Cancers	4	3
Metabolism/Carbohydrate metabolism	114	105
Cellular processes/Cell growth and death	10	9
Organismal systems/Digestive system	1	1
Human diseases/Drug resistance	5	4
Human diseases/Endocrine and metabolic diseases	2	2
Organismal systems/Endocrine system	7	8
Metabolism/Energy metabolism	49	47
Organismal systems/Environmental adaptation	1	1
Genetic information processing/Folding, sorting, and degradation	24	25
Metabolism/Glycan biosynthesis and metabolism	26	26
Human diseases/Immune diseases	1	1
Human diseases/Infectious diseases	9	9
Metabolism/Lipid metabolism	27	27
Environmental information processing/Membrane transport	43	40
Metabolism/Metabolism of cofactors and vitamins	68	64
Metabolism/Metabolism of other amino acids	24	23
Metabolism/Metabolism of terpenoids and polyketides	16	18
Organismal systems/Nervous system	2	2
Human diseases/Neurodegenerative diseases	1	1
Metabolism/Nucleotide metabolism	66	65
Metabolism/Overview	129	126
Genetic information processing/Replication and repair	42	43
Environmental information processing/Signal transduction	18	17
Genetic information processing/Transcription	4	4
Genetic information processing/Translation	86	84
Cellular processes/Transport and catabolism	11	12
Metabolism/Xenobiotics biodegradation and metabolism	14	13
rRNA	9	9
tRNA	57	53
Others	1542	1329

See also Fig. 2.

Supplementary Table S3. Initial sugar composition and concentrations in basal medium containing 1% HMOs as a carbon source.

Sugar[a]	Type I/II[b]	Structure	Conc. (mM)
Fuc		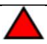	ND
Gal		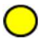	ND
Glc		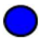	ND
GlcNAc		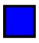	ND
Lac		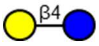	0.46
LNB	Type I	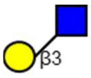	ND
2′-FL		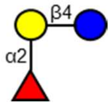	1.9
3-FL		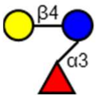	1.5
LDFT		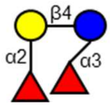	0.44
LNT	Type I	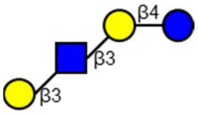	0.85
LN*n*T	Type II	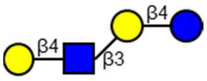	0.34
LNFP I	Type I	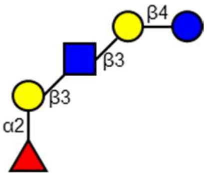	0.46
LNFP II/III	Type I/II	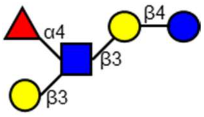 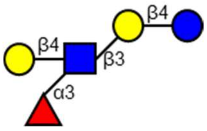	1.0
LNDFH I	Type I	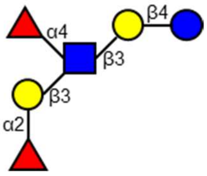	0.68
LNDFH II	Type I	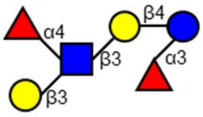	0.33

[a]Sugars were quantified by HPLC following 2-AA labelling, as described in the Methods section.
[b]Type I and type II chains represent Galβ1-3GlcNAc-*O*-R and Galβ1-4GlcNAc-*O*-R, respectively, and are based on blood group chain classification.
ND, not detected.

Supplementary Table S4. Stool sample information

	Age	Sex	Delivery	Feeding
Child-A	4 y	F	vaginal	regular diet
Child-B	5 y	M	vaginal	regular diet
Infant-C	4 m	F	caesarean	breast- and formula- (mixed) feeding
Adult-D	30 y	M	no data	regular diet
Adult-E	39 y	M	no data	regular diet

Supplementary Table S5. Final pH values of faecal cultures incubated in the presence of HMOs or Glc

	Child-A		Child-B		Infant-C		Adult-D		Adult-E		Child-A (+DFJ)	
	HMOs	Glc	HMOs	Glc	HMOs	Glc	HMOs	Glc	HMOs	Glc	HMOs	Glc
None-added	7.1	5.7	7.0	5.8	7.7	5.2	7.1	6.0	7.1	5.8	7.1	5.7
JCM1254	7.0	5.7	7.1	5.7	6.5	5.2	7.4	6.1	7.1	5.8	7.0	5.7
JCM7004	7.0	5.8	7.1	5.8	6.5	5.1	7.4	6.0	7.1	5.8	7.0	5.8
TMC3108	7.0	5.8	7.0	5.8	6.4	5.1	7.4	6.0	7.0	5.8	7.0	5.8
TMC3115	6.9	5.8	7.0	5.8	6.6	5.1	7.4	6.1	7.0	5.8	6.9	5.8

Supplementary Table S6. Primers used for direct sequencing and qPCR analysis.

Target gene	Primer names and sequences			
1,2-α-L-Fucosidase (GH95) (*afcA*)	afcA_up_f [a]	5'-cttgattcttttagtaaacaatg-3'	afcA_dwn_r [a]	5'-gggtttatccgacgggggac-3'
	afcA_f1 [b]	5'-gccgaaggcaagaaggtcatc-3'	afcA_5233_r1 [b]	5'-cgacggtgacctccacgctgg-3'
	afcA_1303_f2 [b]	5'-gacatcatcaaggcagagttc-3'	afcA_4404_r2 [b]	5'-cgcgtacgtggtgcctgcgg-3'
	afcA_2000_r3 [b]	5'-ccgttgtagctggtcgaggatc-3'	afcA_3591_f3 [b]	5'-cttcaccgatgcgaacgccaac-3'
	afcA_f6 [b]	5'-acaccgccgtcaagaaagc-3'	afcA_f7	5'-agccgctcatcgagtatgtg-3'
1,3-1,4-α-L-Fucosidase (GH29) (*afcB*)	afcB_up_f [a]	5'-gtcgaactcttgcatagggtac-3'	afcB_dwn_r [a]	5'-cgtgccatcgccgctccccttg-3'
	afcB_f1 [b]	5'-gtcgaactcttgcatagggtac-3'	afcB_f2 [b]	5'-atggcgtggccggcgagac-3'
	afcB_f3 [b]	5'-aacctccgcgaacttcacc-3'	afcB_f4 [b]	5'-catacgatgcccgatgggt-3'
	afcB_f5 [b]	5'-tgacgtcgatcgagcagctg-3'	afcB_f6 [b]	5'-cgagcccgccgacggatacc-3'
Lacto-*N*-biosidase (GH20) (*lnbB*)	lnbB_up_f [a]	5'-catgcatcttgctaattttg-3'	lnbB_dwn_r [a]	5'-cggcccttcgccaaatatcc-3'
	lnbB_f1 [b]	5'-catgcatcttgctaattttg-3'	lnbB_f2 [b]	5'-tcgccacgaagccgaagta-3'
	lnbB_f3 [b]	5'-tcatcgaatactggtatgg-3'	lnbB_f4 [b]	5'-tccaagaccgtgaacccg-3'
	lnbB_f5 [b]	5'-aacgacgcatggggcctca-3'		
β-Galactosidase III (GH2) (*bbgIII*)	bbgIII_up_f [a]	5'-cgccgtttttgttaatggcatttac-3'	bbgIII_dwn_r [a]	5'-ggggacttagtatgctcgcac-3'
	bbgIII_f1 [b]	5'-gaacaggctgccgtccagcc-3'	bbgIII_f2 [b]	5'-gtggaaccggtcgaagaacg-3'
	bbgIII_f3 [b]	5'-catcctccccgcatggaacg-3'	bbgIII_f4 [b]	5'-ctccgacggcacgtcggacc-3'
	bbgIII_f5 [b]	5'-ccggcaagacgaagatccag-3'	bbgIII_r1 [b]	5'-gccggcgcagaccacgccgc-3'
	bbgIII_f6 [b]	5'-catggaccaccgattacggc-3'	bbgIII_f7 [b]	5'-ggccatcatcgcggatgattc-3'
β-*N*-Acetylglucosaminidase I (GH20) (*bbhI*)	bbhI_up_f [a]	5'-ggagcaaagtcgcaacgaatg-3'	bbhI_dwn_r [a]	5'-cgttattacgtacctgcacg-3'
	bbhI_f1 [b]	5'-gttttaaagtcgagctgaagc-3'	bbhI_f2 [b]	5'-gtgcccgacggctacaccgtc-3'
	bbhI_f3 [b]	5'-gtgcccgacggctacaccgtc-3'	bbhI_f4 [b]	5'-ggactactccatctccaagaag-3'
	bbhI_f5 [b]	5'-ccgttctacgccgagaagac-3'	bbhI_f6 [b]	5'-ggcacgaccaattccgagac-3'
	bbhI_f7 [b]	5'-cagccacgcaggacggtgcc-3'		
GNB/LNB-binding protein (*gltA*)	gltA_up_f [a]	5'-cggtgttgcacgccgtgggttac-3'	gltA_dwn_r [a]	5'-gaaaaacgattccacgagcatg-3'
	gltA_f1 [b]	5'-ccctgacgtacttctacaac-3'		
GNB/LNB phosphorylase (*lnpA*)	lnpAI_up_f [a]	5'-gattaccatcgtccagcgtc-3'	lnpAI_dwn_r [a]	5'-gccttgccccaccgatgggtc-3'
	lnpAI_f1 [b]	5'-gtggactggttcggctgcgc-3'	lnpAI_f2 [b]	5'-gatcatcaacggtggcccgg-3'

qPCR		Sequence	Reference
B. bifidum (species level)	Forward	5'-ccacatgatcgcatgtgattg-3'	(5)
	Reverse	5'-ccgaaggcttgctcccaaa-3'	
Bifidobacterium (genus level)	Forward	5'-agggttcgattctggctcag-3'	(6)
	Reverse	5'-catccggcattaccaccc-3'	

[a]The primers were used for amplification of the respective genes.
[b]The primers were used for sequencing.

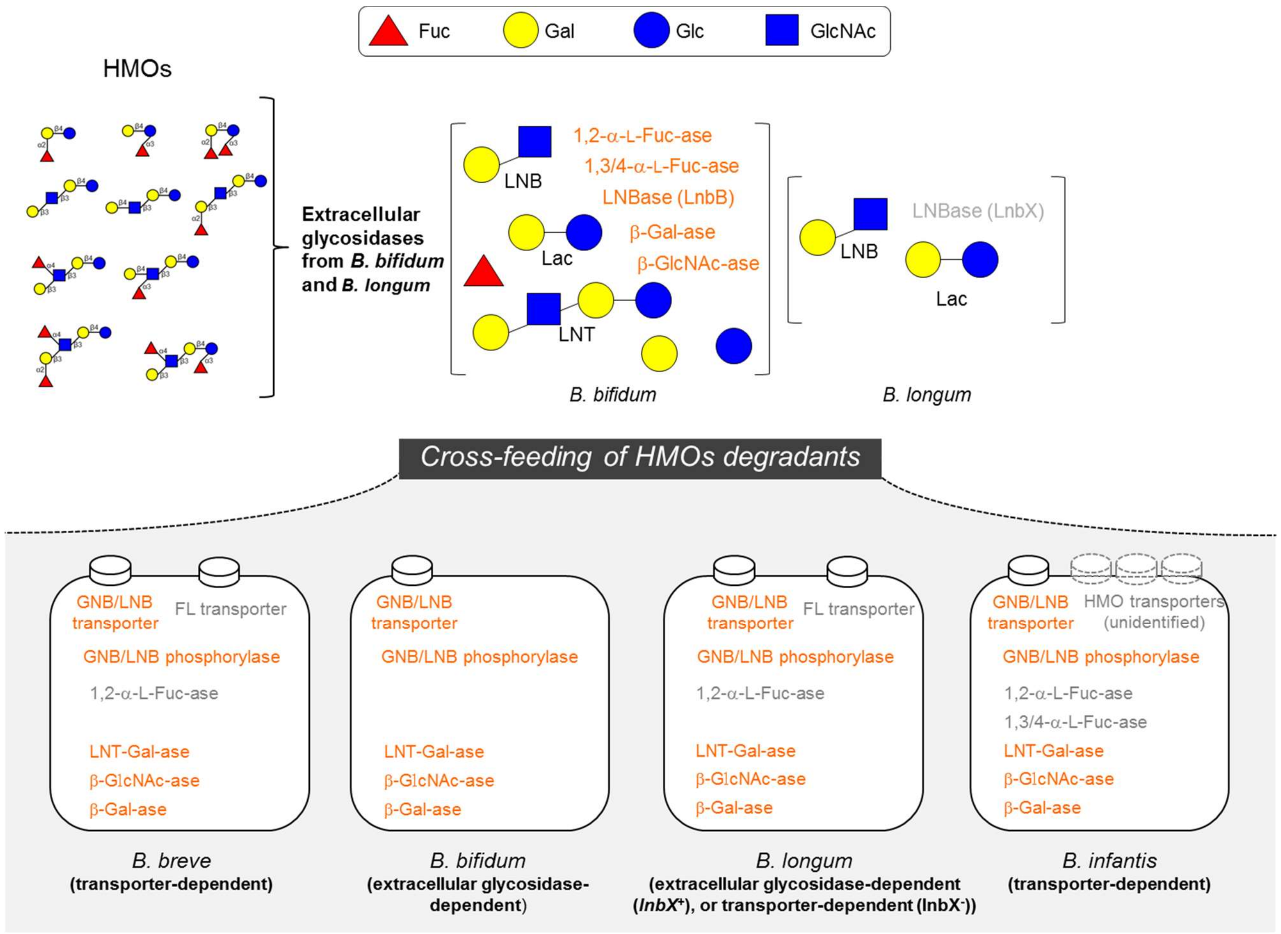

Supplementary Fig. S1. Schematic representation of the HMOs-degrading enzymes in the infant gut-associated bifidobacterial species and the possible cross-feeding strategy within bifidobacterial communities. The enzymes shown in orange are conserved in each species, while the presence of the enzymes shown in grey is strain-dependent.

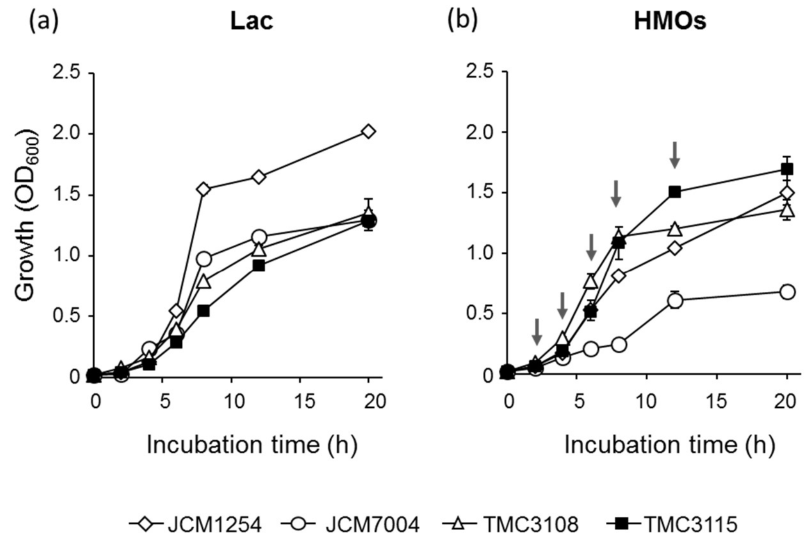

Supplementary Fig. S2. Growth of four *B. bifidum* strains (JCM1254, JCM7004, TMC3108, and TMC3115) in basal medium containing Lac (**a**) or HMOs (**b**) as a carbon source. Overnight culture of each strain was used to inoculate three separate broths, and the growth was monitored by measuring the OD_{600} at the indicated time points. The data are expressed as means ± SD. Samples were collected from HMOs-supplemented cultures at 2, 4, 6, 8, and 12 h post-inoculation (indicated by arrows), and used for analysis of HMOs consumption behaviour of each *B. bifidum* strain by HPLC (see Fig. 3).

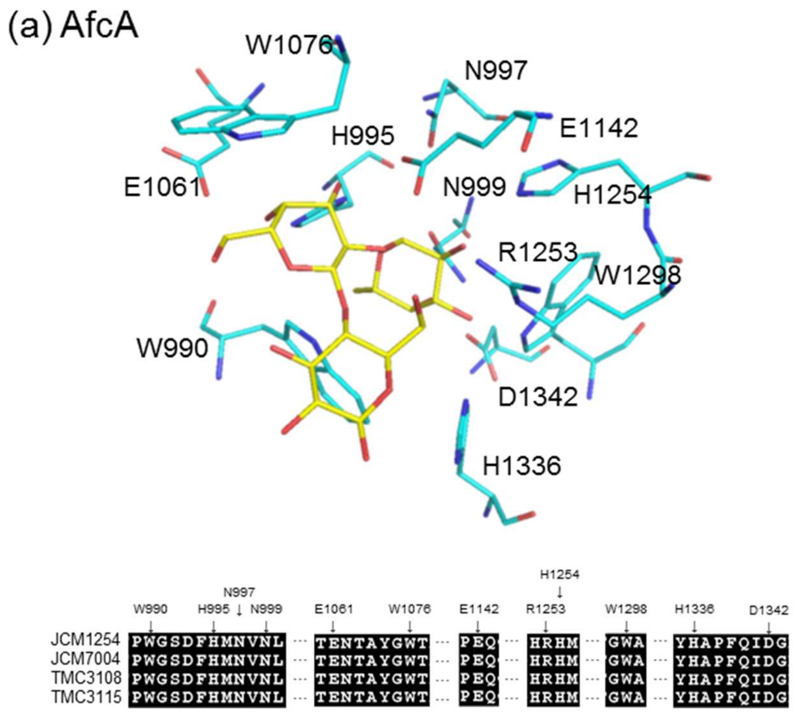

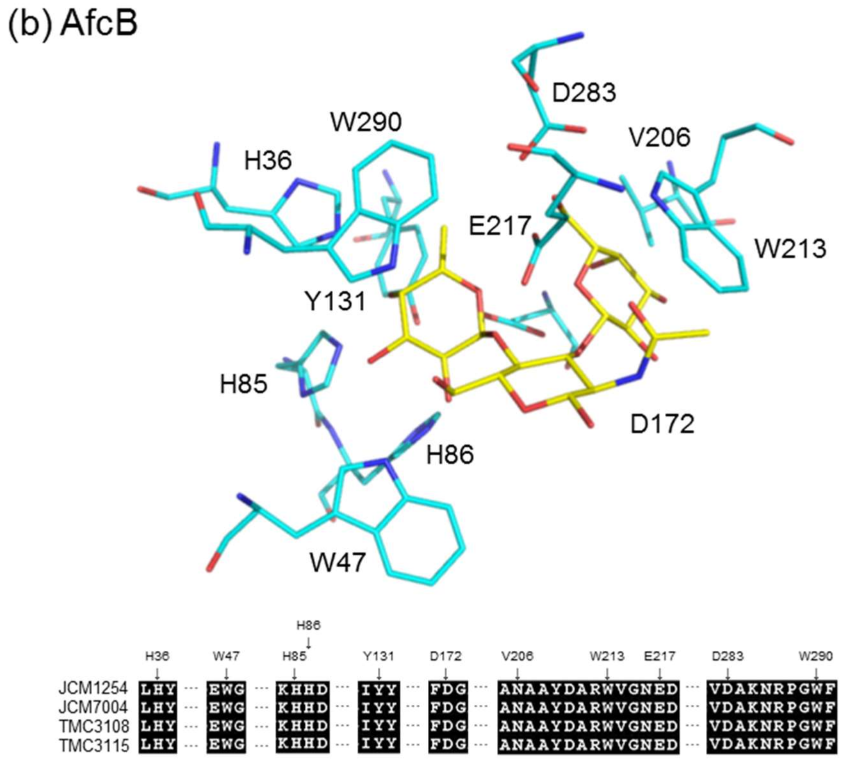

Supplementary Figure S3-*continued*

(c) LnbB

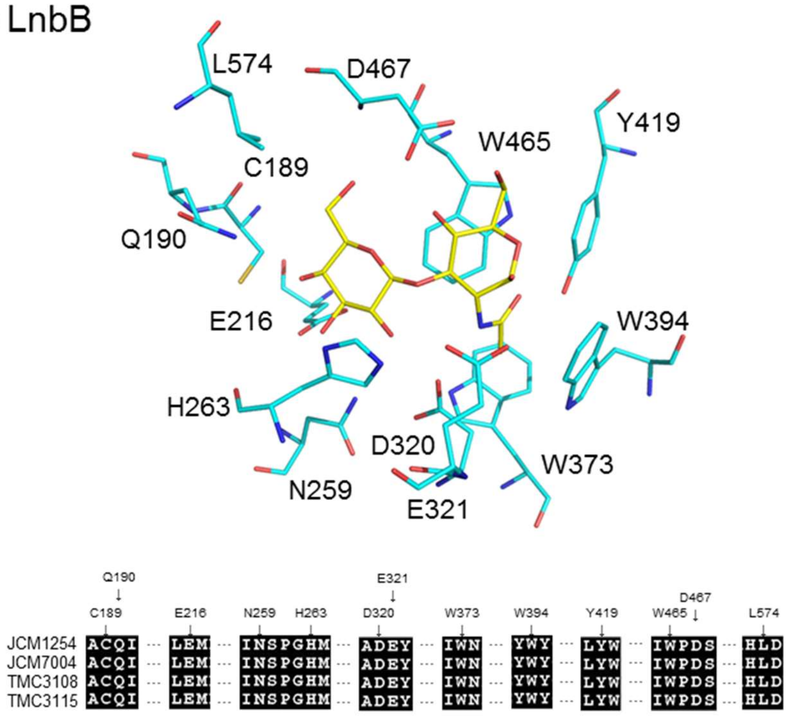

(d) GL-BP

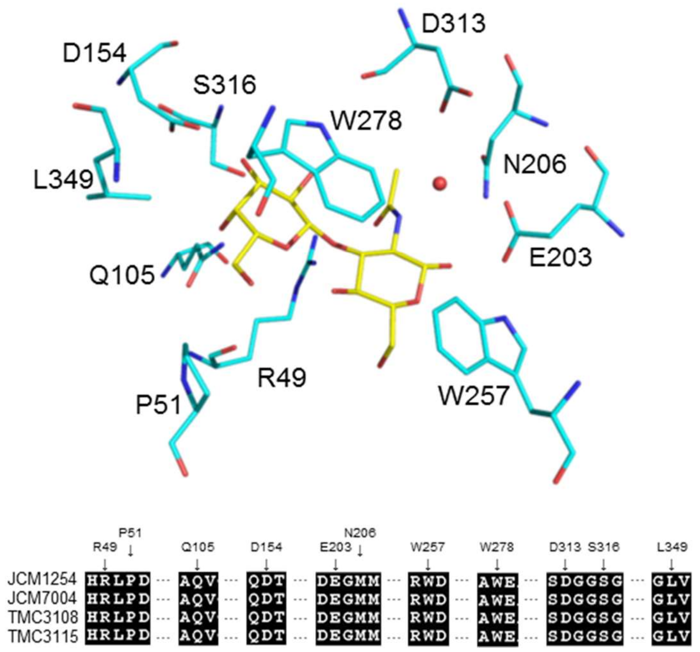

Supplementary Figure S3-*continued*

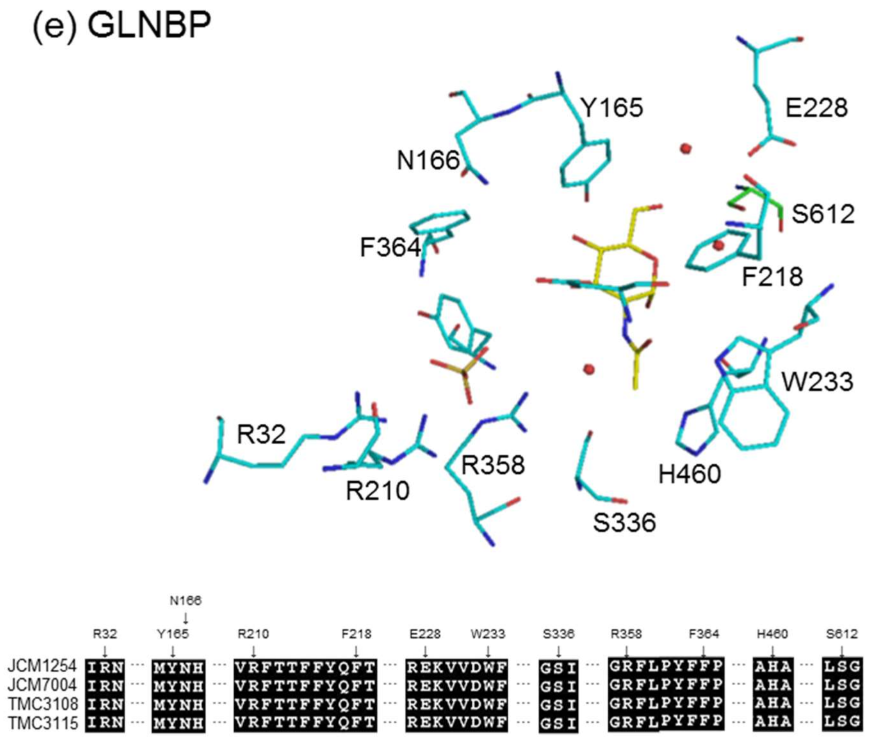

Supplementary Fig. S3. Conservation of HMOs-degrading enzymes in *B. bifidum*. The active site structures of (**a**) 1,2-α-L-fucosidase (AfcA), (**b**) 1,3-1,4-α-L-fucosidase (AfcB), (**c**) lacto-*N*-biosidase (LnbB), (**d**) galacto-*N*-biose/lacto-*N*-biose I-binding protein (GL-BP) of the ABC transporter, and (**e**) galacto-*N*-biose/lacto-*N*-biose I phorphorylase (GLNBP) are shown with their ligands (substrates or products). Alignments of the amino acid residues involved in substrate binding and/or catalysis are shown at the bottom of each panel. These residues are completely conserved in all sequenced *B. bifidum* strains, except for GL-BP from strain JCM1255^{T}. Proteins and ligands (substrates or products) are shown in cyan and yellow, respectively. Water molecules are indicated by red spheres. The numbering of the amino acid residues is based on the structures deposited in Protein Data Bank (2EAC for AfcA; 3UES for AfcB; 4H04 for LnbX; 2ZUW for GLNBP)[7–10]. 2′-FL (2EAD, complexed with AfcA mutant) and Lewis-a trisaccharide (3UET, complexed with AfcB mutant) are docked in the active sites of the respective WT enzyme structures, where deoxyfuconojirimycin (DFJ) was used to overlap with the Fuc moieties of the two substrates[7,8].

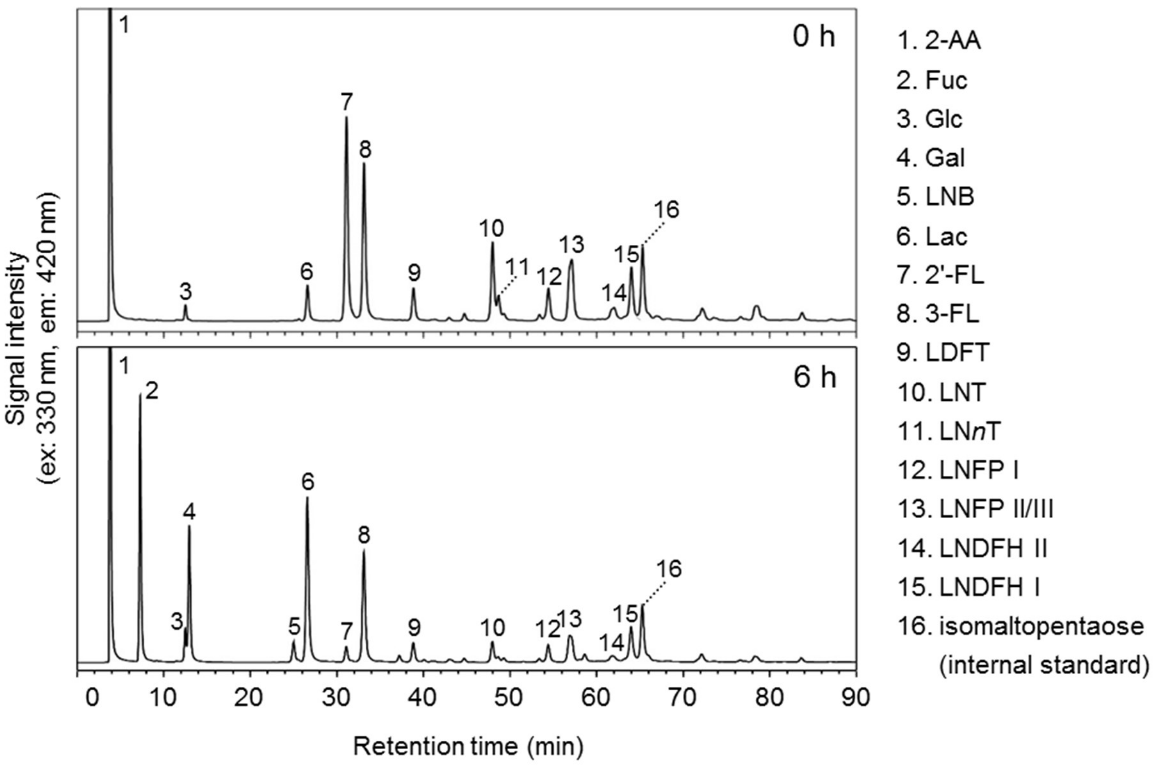

Supplementary Fig. S4. Representative HPLC profiles obtained by analysing spent *B. bifidum* JCM1254 culture medium. Samples were collected at the indicated time points, and the sugars were labelled and analysed by normal-phase HPLC, as described in the Methods section. The numbering of the peaks is based on the retention times, and the corresponding sugars are indicated. See also Supplementary Table S3.

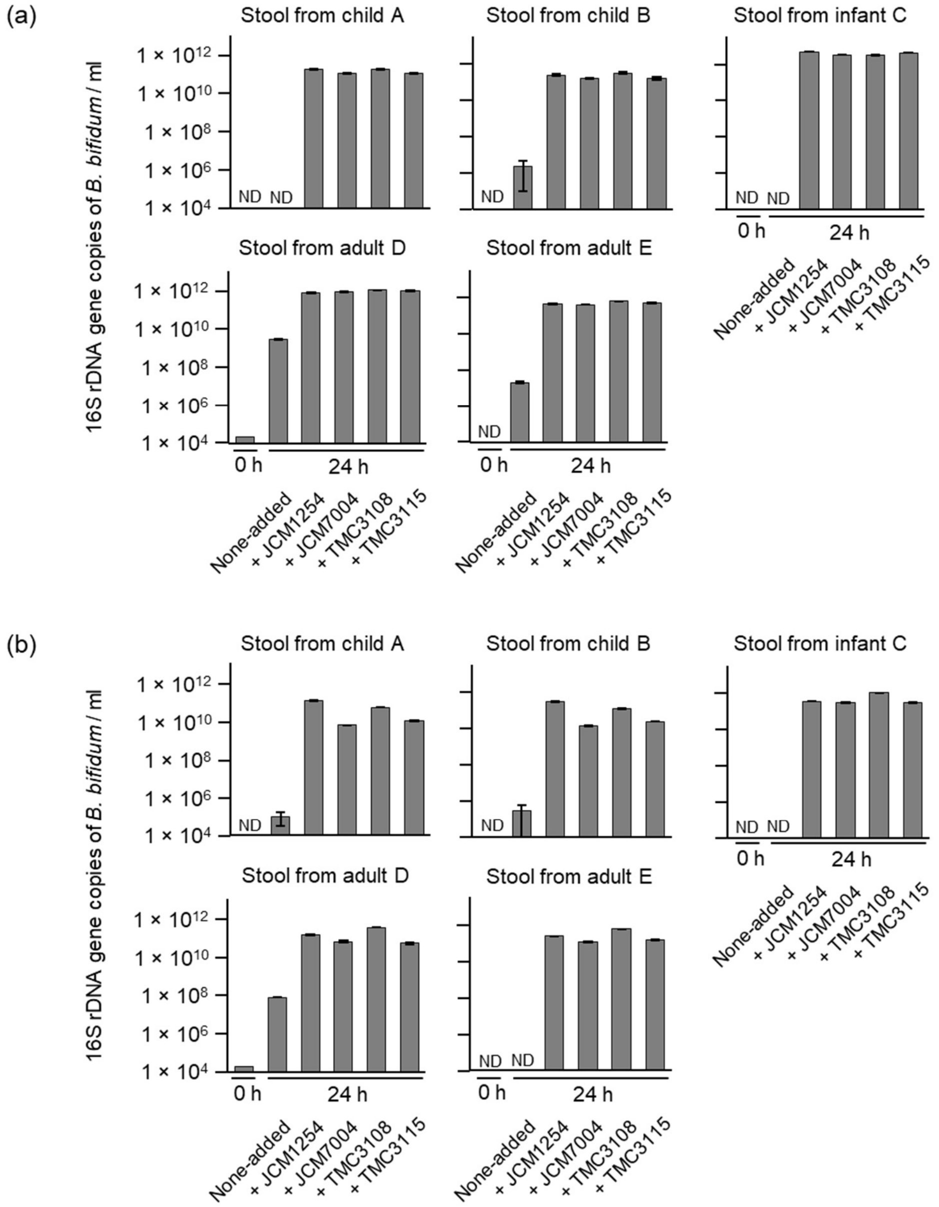

Supplementary Fig. S5. 16S rRNA gene copies attributable to *B. bifidum* in faecal culture supplemented with HMOs or Glc. Stool suspensions from two children (4-year-old female and 5-year-old male), one preweaning infant (caesarean delivered 4-month-old female), and two adults (30-year-old male and 39-year-old male) (see Table S4) were cultured in basal medium containing 1% HMOs (**a**) and Glc (**b**) with and without the addition of four *B. bifidum* strains for 24 h. The abundance of 16S rRNA gene copies corresponding to *B. bifidum* was determined at 0 h and 24 h (dark grey bars) post-inoculation by qPCR, as described in the Methods section. The data are means ± SD of three independent experiments. See also Fig. 5 and Tables S5.

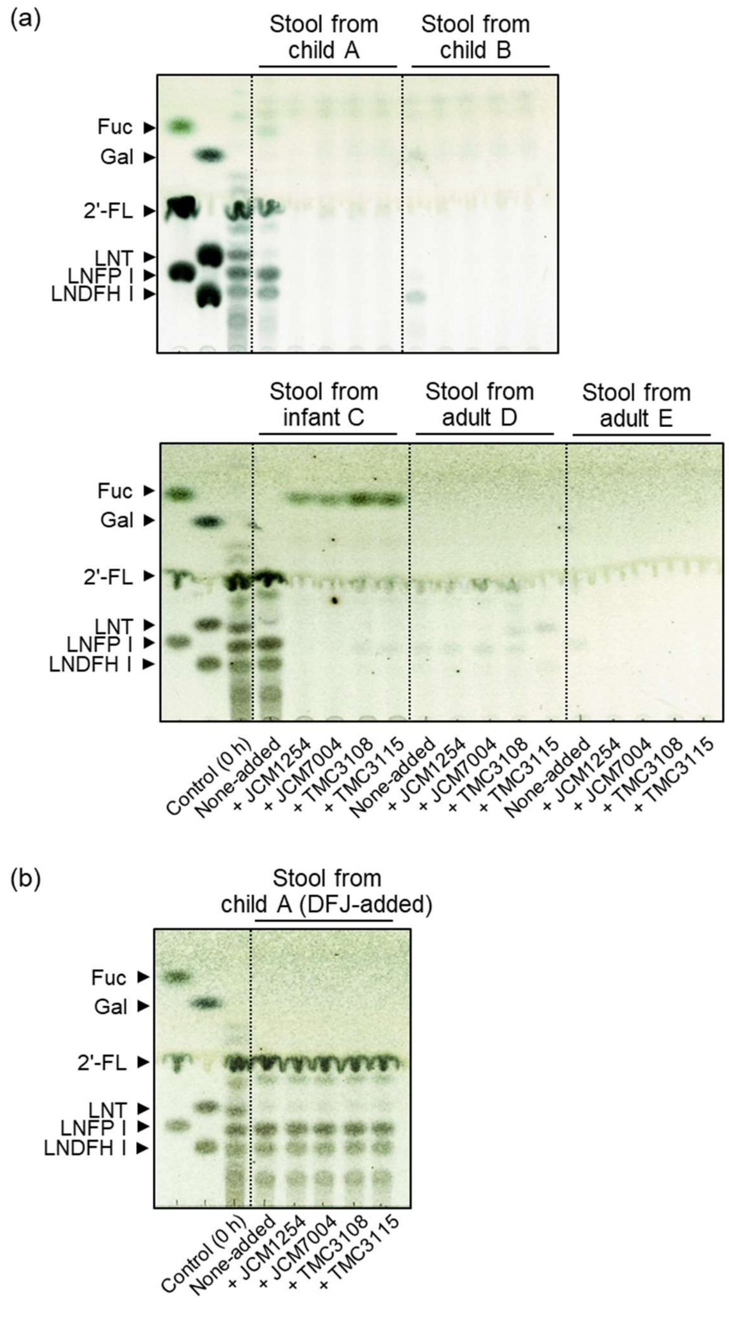

Supplementary Fig. S6. TLC analysis of sugars contained in the spent media. Stool suspensions were incubated for 24 h in the presence of HMOs with and without the addition of *B. bifidum* (a). Stool sample obtained from child A was grown in basal medium supplemented with HMOs in the presence of α-L-fucosidase inhibitor, deoxyfuconojirimycin (DFJ, 500 μM) (b). Fuc, Gal, 2′-FL, LNT, LNFP I, and LNDFH I were used as standard sugars. Culture supernatant collected at time = 0 h was spotted for comparison. Sugars were visualized as described in the Methods section.

(a)

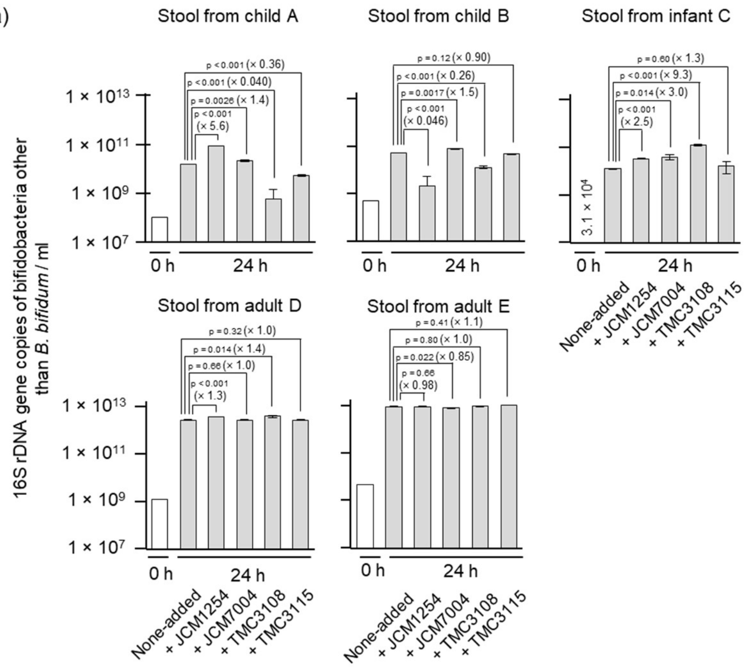

(b)

		None -added	JCM 1254	JCM 7004	TMC 3108	TMC 3115
Child A	Total bacteria (copies/ml) (× 10¹³)	1.4 ± 0.0	2.0 ± 0.0	1.7 ± 0.1	2.0 ± 0.1	1.3 ± 0.0
	Occupancy of other bifidobacterial species in total bacteria (%)	0.11	0.43	0.13	0.0032	0.042
Child B	Total bacteria (copies/ml) (× 10¹³)	1.9 ± 0.1	2.4 ± 0.2	2.0 ± 0.2	2.3 ± 0.1	1.7 ± 0.1
	Occupancy of other bifidobacterial species in total bacteria (%)	0.21	0.0077	0.30	0.045	0.22
Infant C	Total bacteria (copies/ml) (× 10¹³)	0.92 ± 0.02	0.55 ± 0.00	0.92± 0.01	0.76± 0.02	0.68± 0.01
	Occupancy of other bifidobacterial species in total bacteria (%)	0.12	0.50	0.35	1.3	0.20
Adult D	Total bacteria (copies/ml) (× 10¹³)	2.8 ± 0.1	3.1 ± 0.0	2.1 ± 0.1	2.7 ± 0.1	2.3 ± 0.1
	Occupancy of other bifidobacterial species in total bacteria (%)	7.1	8.7	9.5	11	8.4
Adult E	Occupancy of other bifidobacterial species in total bacteria (%)	3.3 ± 0.1	2.8 ± 0.0	2.5 ± 0.1	2.9 ± 0.1	3.0 ± 0.1
	Occupancy of other bifidobacterial species in total bacteria (%)	19	22	22	22	24

Supplementary Fig. S7. Effect of addition of *B. bifidum* to faecal suspensions supplemented with Glc on the abundance of *Bifidobacterium* species other than *B. bifidum* in the culture. (**a**) Stool suspensions from two children (4-year-old female and 5-year-old male), one preweaning infant (caesarean delivered 4-month-old female), and two adults (30-year-old male and 39-year-old male) (see Table S4) were cultured in basal medium containing 1% Glc with and without the addition of four *B. bifidum* strains for 24 h. The abundance of 16S rRNA gene copies corresponding to *Bifidobacterium* species other than *B. bifidum* was determined at 0 h (white bars) and 24 h (grey bars) post-inoculation by qPCR, as described in the Methods section. The data are means ± SD of three independent experiments. Dunnett's test was used to examine the statistical significance. (**b**) Prevalence of *Bifidobacterium* species other than *B. bifidum* in the culture. The total bacterial population was determined as described in the Methods section. Prevalence was calculated by dividing bifidobacterial 16S rRNA gene copies (except for *B. bifidum*) by the total number of bacterial 16S rRNA gene copies. See also Fig. 5 and Tables S5.

(a)

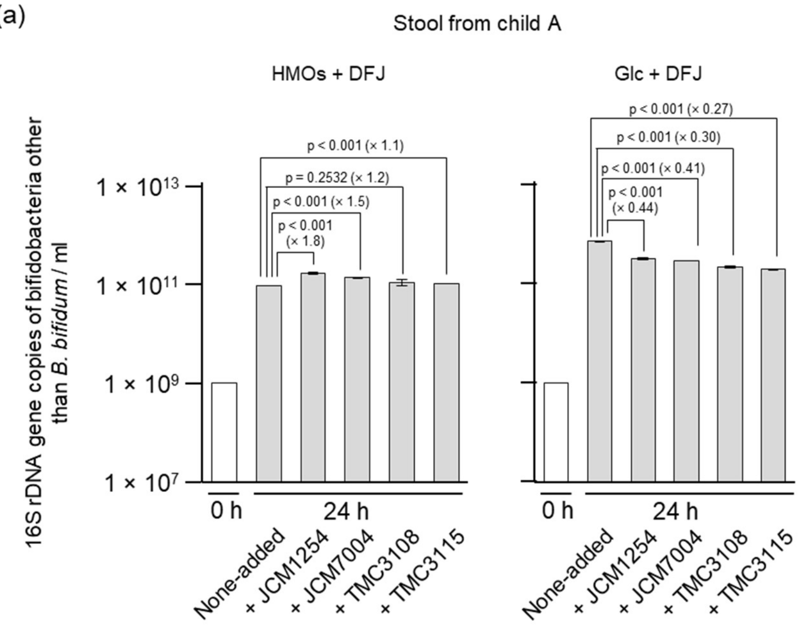

(b)

		None -added	JCM 1254	JCM 7004	TMC 3108	TMC 3115
HMOs + DFJ	Total bacteria (copies/ml) (× 10^{13})	1.1 ± 0.0	1.6 ± 0.1	1.6 ± 0.1	1.6 ± 0.1	1.4 ± 0.0
	Occupancy of other bifidobacterial species in total bacteria (%)	0.86	1.1	0.86	0.69	0.77
Glc + DFJ	Total bacteria (copies/ml) (× 10^{13})	1.3 ± 0.0	1.8 ± 0.1	2.0 ± 0.0	1.4 ± 0.0	1.2 ± 0.1
	Occupancy of other bifidobacterial species in total bacteria (%)	5.7	1.8	1.5	1.6	1.6

Supplementary Fig .S8. Addition of deoxyfuconojirimycin (DFJ) to faecal culture supplemented with HMOs diminished the growth stimulatory effect of *B. bifidum* on other bifidobacterial species. Stool sample obtained from child A was cultured in 1% HMOs-supplemented basal medium with and without the addition of four *B. bifidum* strains for 24 h in the presence of 500 µM DFJ. The abundance of 16S rRNA gene copies corresponding to bifidobacteria other than *B. bifidum* was determined at 0 h and 24 h (grey bars) post-inoculation by qPCR, as described in the Methods section. The data are means ± SD of three independent experiments. See also Fig. 5 and Table S5.

References for supplementary file

1. Morita, H. *et al.* Complete genome sequence of Bifidobacterium bifidum JCM 1255(T) isolated from feces of a breast-fed infant. *J. Biotechnol.* **210,** 66–7 (2015).
2. Turroni, F. *et al.* Genome analysis of Bifidobacterium bifidum PRL2010 reveals metabolic pathways for host-derived glycan foraging. *Proc. Natl. Acad. Sci. U. S. A.* **107,** 19514–9 (2010).
3. Zhurina, D. *et al.* Complete genome sequence of Bifidobacterium bifidum S17. *J. Bacteriol.* **193,** 301–2 (2011).
4. Yu, D. S. *et al.* Complete genome sequence of the probiotic bacterium Bifidobacterium bifidum strain BGN4. *J. Bacteriol.* **194,** 4757–8 (2012).
5. Tannock, G. W. *et al.* Comparison of the compositions of the stool microbiotas of infants fed goat milk formula, cow milk-based formula, or breast milk. *Appl. Environ. Microbiol.* **79,** 3040–3048 (2013).
6. Ahmed, S. *et al.* Mucosa-associated bacterial diversity in relation to human terminal ileum and colonic biopsy samples. *Appl. Environ. Microbiol.* **73,** 7435–42 (2007).
7. Nagae, M. *et al.* Structural basis of the catalytic reaction mechanism of novel 1,2-alpha-L-fucosidase from Bifidobacterium bifidum. *J. Biol. Chem.* **282,** 18497–509 (2007).
8. Sakurama, H. *et al.* 1,3-1,4-α-L-fucosynthase that specifically introduces Lewis a/x antigens into type-1/2 chains. *J. Biol. Chem.* **287,** 16709–19 (2012).
9. Ito, T. *et al.* Crystal structures of a glycoside hydrolase family 20 lacto-N-biosidase from Bifidobacterium bifidum. *J. Biol. Chem.* **288,** 11795–11806 (2013).
10. Hidaka, M. *et al.* The crystal structure of galacto-N-biose/lacto-N-biose I phosphorylase: a large deformation of a TIM barrel scaffold. *J. Biol. Chem.* **284,** 7273–83 (2009).
